# Genomic GPS: using genetic distance from individuals to public data for genomic analysis without disclosing personal genomes

**DOI:** 10.1186/s13059-019-1792-2

**Published:** 2019-08-27

**Authors:** Kunhee Kim, Hyungryul Baik, Chloe Soohyun Jang, Jin Kyung Roh, Eleazer Eskin, Buhm Han

**Affiliations:** 1 Department of Biomedical Sciences, Asan Medical Center, AMIST, University of Ulsan College of Medicine, Seoul, South Korea; 2 Department of Mathematical Science, KAIST, Daejeon, South Korea; 3 Department of Medical Sciences, Seoul National University College of Medicine, Seoul, South Korea; 4 Department of Convergence Medicine, University of Ulsan College of Medicine, Asan Medical Center, Seoul, South Korea; 5 Department of Computer Science and Human Genetics, University of California Los Angeles, Los Angeles, CA USA; 6 Department of Computational Medicine, University of California Los Angeles, Los Angeles, CA USA

**Keywords:** Multilateration, Genetic distance, Personal genome, Data sharing, Privacy protection

## Abstract

Genomic global positioning system (GPS) applies the multilateration technique commonly used in the GPS to genomic data. In the framework we present here, investigators calculate genetic distances from their samples to reference samples, which are from data held in the public domain, and share this information with others. This sharing enables certain types of genomic analysis, such as identifying sample overlaps and close relatives, decomposing ancestry, and mapping of geographical origin without disclosing personal genomes. Thus, our method can be seen as a balance between open data sharing and privacy protection.

**Electronic supplementary material:**

The online version of this article (10.1186/s13059-019-1792-2) contains supplementary material, which is available to authorized users.

## Main text

It is crucial to balance privacy protection and data sharing in genomics [1–3]. Full disclosure of genomic data benefits the research community through productive data reuse but increases the chances of privacy breaches. Full closure, by contrast, ensures privacy but discourages collaborative science.

Here we present a method called *genomic GPS* that aims to achieve a balance between data sharing and privacy protection. It allows sharing of information to a degree sufficient for approximating relative genetic distance of an individual from either another individual or a group. Identification of closer relatives and population genomic analyses, such as ancestry decomposition and geographical origin mapping, are possible. Importantly, though, the shared information conceals individual genotypes, making it extremely difficult to reconstruct the personal genomes.

**Fig. 1:**
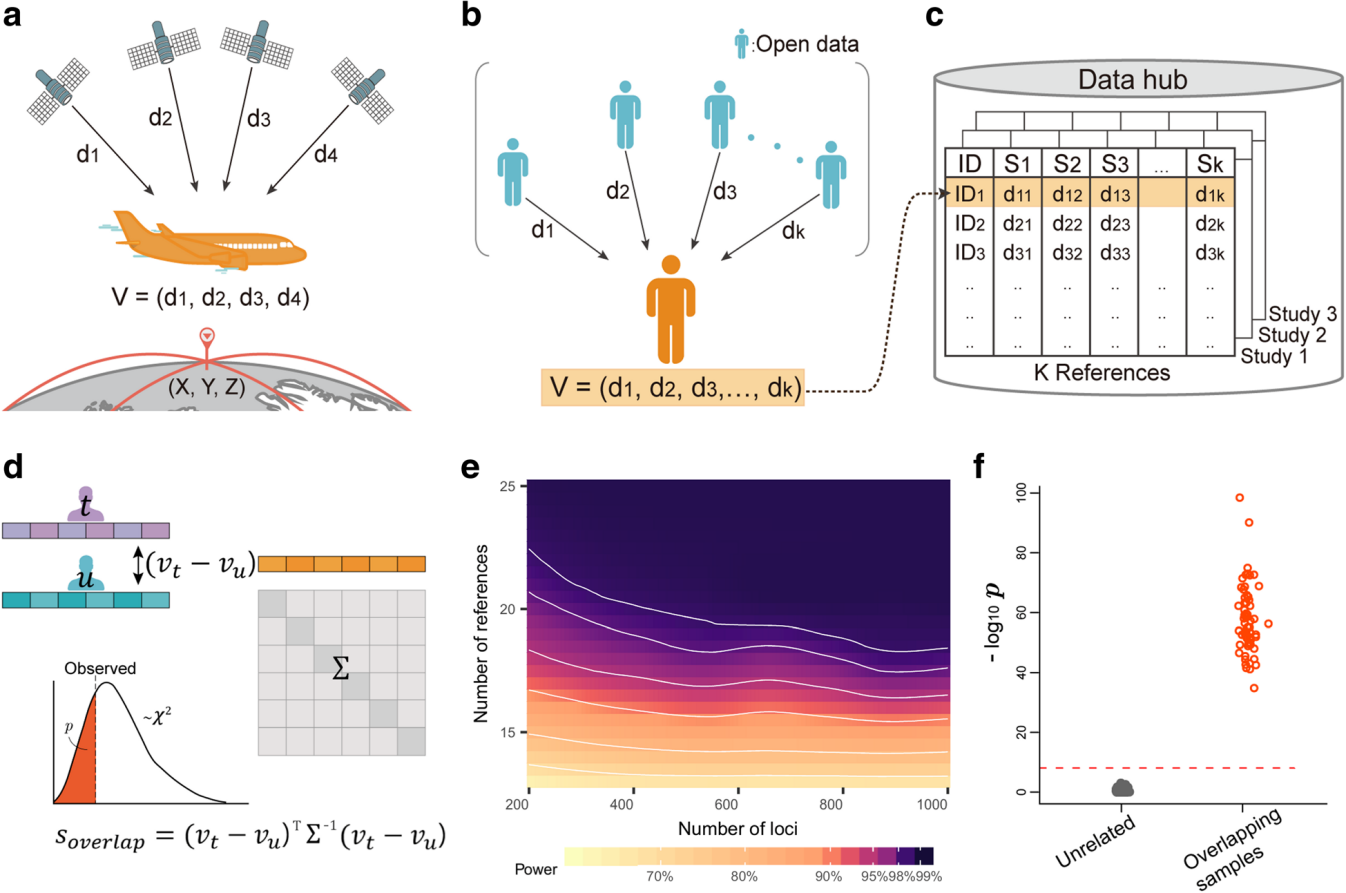
Genomic GPS and its application to sample overlap detection. **a** The concept of conventional GPS. Distances to satellites are used to compute an aircraft’s location. **b** The concept of genomic GPS. The genetic distances of an individual to reference individuals in public datasets are calculated to create a distance vector. **c** Distance vectors can be shared, for example, by using a public data hub. **d** Sample overlap detection using distance vectors. The distance vectors of two individuals are compared using a statistic that follows a *χ*^2^ distribution. **e** The power of the sample overlap detection method as a function of the number of loci and the number of reference individuals used to calculate the genetic distances. **f** *P* values of the sample overlap detection method for overlapping pairs and unrelated pairs in the simulations using the WTCCC data

Our method builds upon *multilateration*, a localization technique for wireless sensor networks in which spatial coordinates of a node with an unknown position are inferred by measuring the distances from the node to several reference nodes at known positions [4]. For example, in the GPS navigation system of an aircraft, the distances from the aircraft to satellites are calculated from time lags in transmitted radio signals. These distances are then used to calculate the aircraft’s position (Fig. 1a).

To apply multilateration in a genomic context, we first evaluated this technique’s characteristics in a multidimensional space. We derived a mathematical proof showing that in *N*-dimensional space, and with *K* reference nodes with known positions, an unknown node’s coordinates can be unequivocally identified if *K* > *N* (Additional file 1: Supplementary Note). Perhaps more importantly, we derived another proof showing that an unknown node’s coordinates can never be exactly specified if *K* < *N* − 1 (Additional file 1: Supplementary Note). This was encouraging because it suggested that the distances to known nodes convey limited information under this condition and can be safely shared without disclosing the actual location.

Encouraged by this proof, we applied multilateration to genomic data. We considered an individual’s genotype data to be a node in *N*-dimensional space where each coordinate represents each of *N* polymorphic loci. In this space, the pairwise Euclidean distance between nodes represents the genetic distance between individuals (Fig. 1b). We then measured genetic distances from that individual to *K* reference individuals in open datasets such as 1000Genomes [5]; these datasets are analogous to satellites with known positions. We call the length-*K* vector of distances the “*distance vector*”. The core idea of our approach is to share a distance vector that would allow certain types of genomic analysis without disclosing the personal genome data (Fig. 1c).

If we imagined that genotypes were real numbers, it is theoretically impossible to reconstruct the genotypes as long as *N* ≫ *K*, as shown by our proof. Unfortunately, genotype data resides in a very restricted space, {0, 1, 2}^*N*^. Nevertheless, the search space is still large enough to prevent data reconstruction in practice. We designed a greedy algorithm that tries to reconstruct the genotype data given a distance vector and reference data (Additional file 1: Supplementary Note) and applied it to simulated data. To avoid local optima, we allowed multiple restarts of the algorithm to find the best possible solution. The predicted genotypes were not much better than a coarse prediction based on allele frequency (Additional file 1: Figure S1). These empirical simulations showed that it was impractical to reconstruct genotypes from a distance vector.

Sharing of distance vectors facilitates several applications. First, we can use the similarity of two distance vectors to find sample overlaps or close relatives. Intuitively, if genomes from two individuals resemble each other, their distance vectors will also be similar. To systematically interpret the similarity in distance vectors, we designed the following statistic. Let *X*_*t*, *n*_ ∈ {0, 1, 2} be the reference allele count of individual *t* at SNP *n*. The squared Euclidean distance between individuals *t* and *u* is ${D}_{t,u}=\sum \limits_{n=1}^N{\left({X}_{t,n}-{X}_{u,n}\right)}^2$. Let *D*_*t*, *k*_ be the distance between *t* and reference individual *k*. Given *K* reference individuals, the distance vector of individual *t* is *v*_*t*_ = (*D*_*t*, 1_, *D*_*t*, 2_…, *D*_*t*, *K*_). Then, we define a statistic that compares two distance vectors, *v*_*t*_ and *v*_*u*_:

$$ {s}_{\mathrm{overlap}}={\left({v}_t-{v}_u\right)}^T{\Sigma}^{-1}\left({v}_t-{v}_u\right) $$

where Σ is the covariance matrix of *v*_*t*_ − *v*_*u*_. We showed (Additional file 1: Supplementary Note) that the (*i*, *j*)th element of Σ is

$$ {\Sigma}_{ij}=\left\{\begin{array}{c}\ \sum \limits_{n=1}^N24{p}_n^4-48{p}_n^3+20{p}_n^2+4{p}_n\kern0.96em \left(i=j\right)\\ {}\sum \limits_{n=1}^N-8{p}_n^4+16{p}_n^3-12{p}_n^2+4{p}_n\kern0.96em \left(i\ne j\right)\end{array}\right. $$

where *p*_*n*_ is the population allele frequency of SNP *n*. The statistic *s*_overlap_ follows a *χ*^2^ distribution with *K* degrees of freedom if *t* and *u* are unrelated (Fig. 1d); thus, we can test whether two individuals are related by calculating a *P* value from the lower tail. The false positive rate was well controlled (Additional file 1: Table S1 and Figure S2), and high power was achievable for reasonable numbers of *N* and *K* (Fig. 1e). This statistic can be useful if researchers at different institutions want to check whether there are overlapping individuals in their samples, because sample overlaps can contaminate the result of aggregate studies such as meta-analyses. Instead of the squared Euclidean distance, one can also use genetic relatedness metric as the measure of genetic distance. Given the standardized allele count ${\overline{X}}_{t,n}=\left({X}_{t,n}-2{p}_n\right)/\sqrt{2{p}_n\left(1-{p}_n\right)}$, the genetic relatedness between individuals *t* and *u* is ${G}_{t,u}=\frac{1}{N}{\sum}_{n=1}^N{\overline{X}}_{t,n}{\overline{X}}_{u,n}$ [6]. Using genetic relatedness, the results were similar (Additional file 1: Supplementary Note and Figure S3). We performed real data-based analysis using the Wellcome Trust Case Control Consortium (WTCCC) data [7] by designing studies with overlapping samples (Additional file 1: Supplementary Note). Our method could detect overlapping samples with perfect sensitivity and specificity (Fig. 1f), when using the 1000Genomes data [5] as reference.

We then examined whether close relatives were also distinguishable using our statistic. We simulated different degrees of relatives and predicted the true relationship for a given pair using our statistic. Among first-degree relatives, 79% were correctly predicted as first degree (Additional file 1: Figure S4 and S5). Relatives beyond the first degree were less distinguishable, where 39% and 21% of second- and third-degree relatives were correctly predicted, respectively. In sum, distance vectors contain sufficient information to determine overlapping samples and to give clues for close relatives, which can be useful for certain types of analyses. However, such a disclosure could be considered a leak of information in some situations. In those situations, alternatives such as secure hashing [3] can be considered for detecting sample overlaps.

The second application for sharing a distance vector is population genomic analyses. The distance vector contains information that can infer the genetic spatial structure of individuals. Recent studies showed that genetic data enabled the geographical origin of an individual to be located on a two-dimensional (2D) map [8, 9]. Novembre et al. [8] applied principal component analysis (PCA) to the genomic data of 3192 Europeans from 36 countries (the POPRES dataset [10]), where the two main principal components (PC) matched the geographical map of Europe with great accuracy. We designed a procedure that converts a distance vector into an approximate position in the PC space (Additional file 1: Supplementary Note). Consider that we have *K* reference individuals. We first apply eigendecomposition to their genetic relatedness matrix (GRM) to obtain the top two eigenvectors (PCs) in a 2D space, $\mathcal{P}$. Given a target individual, we want to approximate its position in $\mathcal{P}$. Suppose that we have the target’s distance vector to *K* references based on the genetic relatedness metric. Then, we can construct the GRM of the *K* + 1 individuals (the references and the target) by appending the distance vector to the GRM. We decompose this (*K* + 1) × (*K* + 1) GRM to obtain a PC map of *K* + 1 individuals in a new 2D space, $\mathcal{P}^{\prime }$. The positions of the *K* references in $\mathcal{P}^{\prime }$ are not identical to their positions in $\mathcal{P}$, because adding one more datapoint in PCA can distort the positions of the other points (Additional file 1: Figure S6). Because of this subtle difference, in order to project the target’s point from $\mathcal{P}^{\prime }$ to $\mathcal{P}$, we apply another layer of “multilateration”. Using the map in $\mathcal{P}^{\prime }$, we calculate the 2D Euclidean distances between the target and the references to create a distance vector. Using the standard multilateration technique, this distance vector can be used to map the target’s position in $\mathcal{P}$ by the least-square minimization [4]. After repeating this procedure for each target, the approximated PC map of all target individuals is obtained by removing reference datapoints from $\mathcal{P}$.

**Fig. 2:**
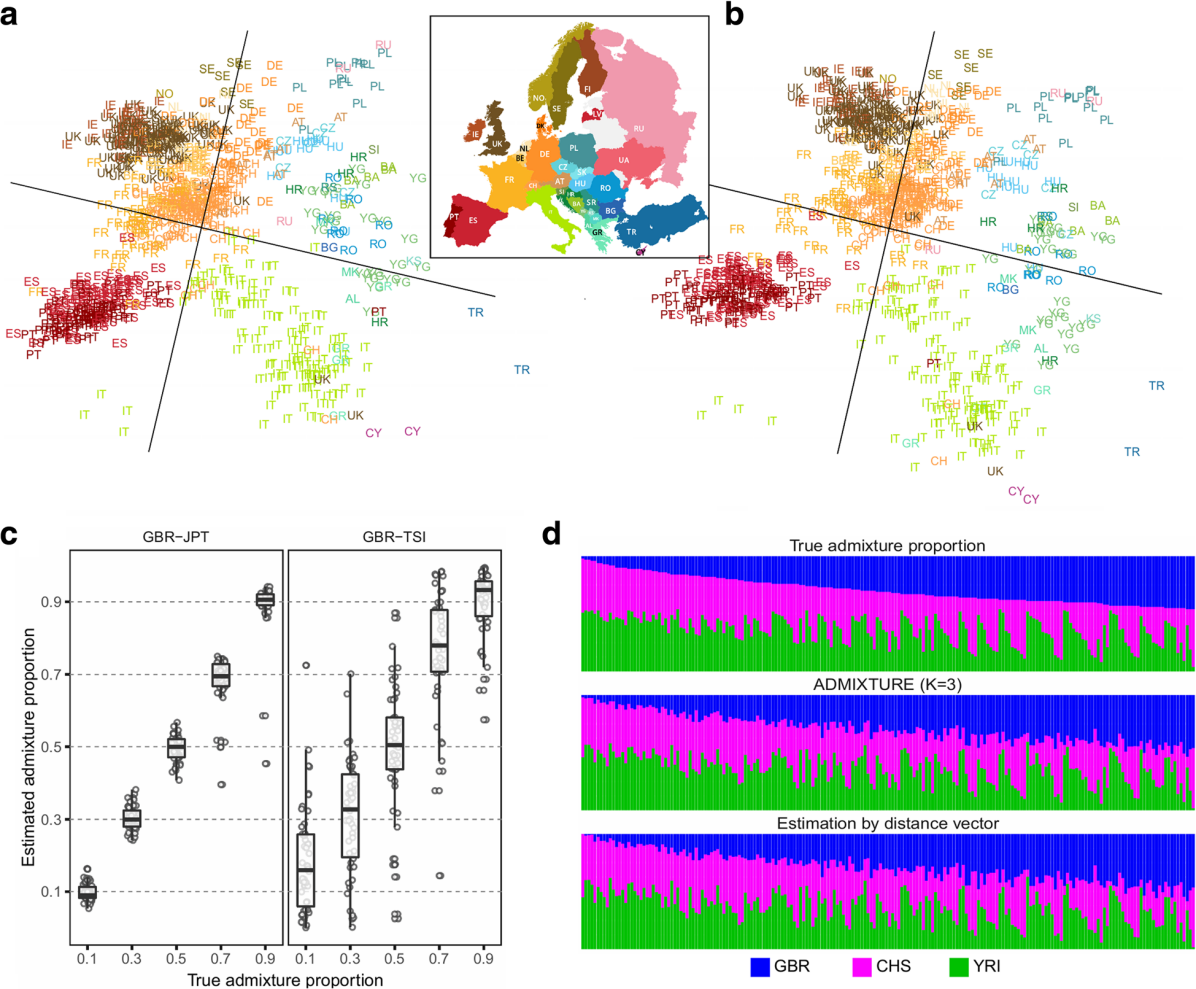
Population genomic applications of distance vectors. **a** Two-dimensional mapping of the Europeans in the POPRES data using only distance vectors. We mapped a subset (60%) of the POPRES individuals and used the rest of the individuals (40%) as references. See Additional file 1: Table S2 for the abbreviated population names. **b** Mapping result of the same individuals using actual genomic data (the top two PCs). **c** Estimation of admixture proportion using distance vectors. We simulated admixed individuals from two distant populations (GBR: British in England and Scotland and JPT: Japanese in Tokyo, Japan) and two close populations (GBR and TSI: Toscani in Italia) using the 1000Genomes data. **d** Admixture of three populations (GBR, CHS: Southern Han Chinese, and YRI: Yoruba in Ibadan, Nigeria). The proportions were estimated using distance vectors and ADMIXTURE

To evaluate the performance of our method, we used the POPRES data [10] (Additional file 1: Table S2) using 60% of the individuals as samples and 40% as references (Additional file 1: Supplementary Note). The mapping of the origins of the samples using our method (Fig. 2a) closely resembled the PC mapping based on actual genotype data (Fig. 2b). The output image resembled the geographic map of Europe, with geographically adjacent populations found near to each other and geographically distant populations found far apart. We then tried to map the POPRES data using the 1000Genomes samples [5] as reference data. Overall, the approximate locations of the populations were similar to the European map (Additional file 1: Figure S7). However, the distinction between the Eastern/Russian populations and the Central European populations was unclear, possibly because there is sparse data from these populations in this reference dataset [5].

Another application for sharing distance vectors in population genomics is the inference of an individual’s ethnic admixture proportions. We designed a procedure to estimate the admixture proportion of an individual only using the distance vector (Additional file 1: Supplementary Note). The idea is to approximate the location of a target individual in the PC map of the multiple reference populations. We measure the Euclidean distance of the individual to the centroid of each candidate population and estimate the ancestry proportions as being inversely proportional to these distances. Using the 1000Genomes data, we simulated admixed individuals from two populations, gradually varying the proportions. When the two populations were genetically distant (European and Asian), the estimated proportion was close to the true proportion (*r*^2^ = 0.98, Fig. 2c). When the two populations were genetically close (two European countries), the estimation was less accurate but showed high correlation to the true proportion (*r*^2^ = 0.86, Fig. 2c). We then combined data for three populations (European, Asian, and African) in varying proportions. For comparison, we applied an existing method, ADMIXTURE [11], which uses actual genotype data (Additional file 1: Supplementary Note). Both ADMIXTURE and our method gave estimations that were highly concordant with the true proportions (Fig. 2d).

We have presented a novel technique that applies multilateration to genomic data. Our method allows sharing distance vectors with other investigators or institutions, enabling certain types of genomic analysis while making it difficult to reconstruct the personal genomes. We expect that our approach will find interesting applications in the future in addition to those described herein.

## Methods

See Additional file 1: Supplementary Note for additional methods not described above**.**

## Additional file

Additional file 1: Supplementary Note, Tables S1, S2 and Figures. S1-S12. (PDF 3657 kb)

## Data Availability

The Genomic GPS is available at https://github.com/hanlab-SNU/GenomicGPS [12] under the MIT license (Software DOI: 10.5281/zenodo.3255141 [13]). Genotype data for 1000Genomes Phase 1 was downloaded from http://www.cog-genomics.org/plink/1.9/resources. Imputed haplotype data of 1000Genomes Phase 3 was downloaded from ftp://ftp.1000genomes.ebi.ac.uk/vol1/ftp/release/20130502/ [5]. WTCCC data used for sample overlap simulation can be accessible via EGA accession number EGAD00000000001 for 1958 British Birth Cohort and EGAD00000000008 for Type 1 Diabetes (T1D) samples (https://www.ebi.ac.uk/ega/datasets) [7]. POPRES data used for PC map analysis is accessible via dbGaP Study accession number phs000145.v4.p2 (https://www.ncbi.nlm.nih.gov/projects/gap/cgi-bin/study.cgi?study_id=phs000145.v4.p2) [10]. Detailed description of datasets used in this research can be found in Additional file 1: Supplementary Note.
