# Supplementary material for: Genomic GPS: using genetic distance from individuals to public data for genomic analysis without disclosing personal genomes
Source: Genome Biol. 2019 Aug 27;20:175. doi: 10.1186/s13059-019-1792-2 (PMC6712716; doi:10.1186/s13059-019-1792-2)

Supplementary Materials for "Genomic GPS: using genetic distance from individuals to public data for genomic analysis without disclosing personal genomes"

Kunhee Kim[1,3,*], Hyungryul Baik[2,*], Chloe Soohyun Jang[3], Jin Kyung Roh[4], Eleazer Eskin[5], and Buhm Han[3]

[1] Department of Biomedical Sciences, Asan Medical Center, AMIST, University of Ulsan College of Medicine, Seoul 05505, South Korea.

[2] Department of Mathematical Science, KAIST, Daejeon, South Korea

[3] Department of Medical Sciences, Seoul National University College of Medicine, Seoul, South Korea

[4] Department of Convergence Medicine, University of Ulsan College of Medicine, Asan Medical Center, Seoul, South Korea

[5] Department of Computer Science and Human Genetics, University of California Los Angeles, Los Angeles, CA, USA

Table of contents

SUPPLEMENTARY NOTE **3**

1 BASIC CONCEPT OF THE METHOD **3**

2 SAMPLE OVERLAP DETECTION **3**

2.1 Method 3
2.2 Covariance matrix Σ 3
2.3 P-value 11
2.4 Significance threshold 11
2.5 False positive rate and power simulation 12
2.6 Real data simulation using WTCCC 12
2.7 Identification of relatives 14
2.8 Genetic relatedness metric 14

3 PRINCIPAL COMPONENT MAP CONSTRUCTION **15**

3.1 Method 15
3.2 Real data analysis using POPRES 16

4 ANCESTRY ESTIMATION **17**

4.1 Method 17
4.2 Two population simulation 18
4.3 Three population simulation 18

5 UNIDENTIFIABILITY **19**

5.1 Mathematical proofs 19
5.2 Simulations 22
5.3 Complexity analysis 25

6 DATASETS USED IN THE ANALYSIS **25**

7 SOFTWARE IMPLEMENTATION **26**

REFERENCES **28**

SUPPLEMENTARY TABLES **30**

SUPPLEMENTARY FIGURES **32**

Supplementary Note

1 Basic concept of the Method

Suppose that an individual's genomic sequence has N loci. A common form of the locus is single nucleotide polymorphism (SNP), where each locus can have a value of 0, 1, or 2 (the count of the reference allele). Given N SNPs, we define an N-dimensional space, where an individual's SNP data specifies the individual's position. The distance between two individuals in this space represents the genetic distance between the two. Assume that we have a target individual t whose position in this space is unknown and a reference set of multiple (K) individuals with known positions, such as samples from the HapMap [1] or 1000Genomes [2]. We can calculate the distances between the target individual t and those of the reference individuals to obtain a length-K vector, v_t, which we call a distance vector. The ith element of the distance vector represents the distance between t and the ith reference individual (**Fig. 1b**). A distance vector can be shared among institutions or researchers for a number of purposes, as discussed in the main article, without disclosing the individual's genotype data (**Fig. 1c**).

2 Sample overlap detection

2.1 Method

Distance vectors can be used for detecting sample overlaps. Consider two target individuals t and u whose distance vectors to K satellites (v_t and v_u) are known. To detect if t and u are a sample overlap, we calculate a statistic

$$s_{\text{overlap}} = (v_t - v_u)^T \Sigma^{-1} (v_t - v_u), \tag{1}$$

where Σ is the covariance matrix of $v_t - v_u$. Under the condition that loci are independent, our statistic follows a χ^2 distribution with K degrees of freedom (df) under the null hypothesis that the two individuals are unrelated.

For measuring the distance between the two individuals, differing metrics can be used. Below, we use the squared Euclidean distance. If we use the squared Euclidean distance, Σ can be analytically calculated. However, a different metric such as the genetic relatedness can also be used for this statistic, which we describe later.

Let $X_{t,n} \in \{0,1,2\}$ be the reference allele count of individual t at SNP n. The squared Euclidean distance between individuals t and u is $D_{t,u} = \sum_{n=1}^{N} (X_{t,n} - X_{u,n})^2$.

2.2 Covariance matrix Σ

Below we derive the closed form of the covariance matrix, step by step.

Two individuals.

Suppose that we have two individuals A and B. Suppose that we have a single locus (N=1). For simplicity, we will use the same letters (A and B) to refer to the reference allele count of A and B. Given that A and

B can have a value of 0, 1, or 2, the squared Euclidean distance, $(A-B)^2$, can have a value of 0, 1, or 4. Under the null hypothesis that the two individuals are unrelated, each individual has 0, 1, or 2 with probability p^2, $2p(1-p)$, and $(1-p)^2$ where p is the population frequency of the non-reference allele. If we enumerate all possible cases and their probabilities,

A	B	$(A-B)^2$	Probability
0	0	0	p^4
0	1	1	$2p^3(1-p)$
0	2	4	$p^2(1-p)^2$
1	0	1	$2p^3(1-p)$
1	1	0	$4p^2(1-p)^2$
1	2	1	$2p(1-p)^3$
2	0	4	$p^2(1-p)^2$
2	1	1	$2p(1-p)^3$
2	2	0	$(1-p)^4$

We can summarize the table with respect to $(A-B)^2$,

$(A-B)^2$	Probability
0	$p^4 + 4p^2(1-p)^2 + (1-p)^4$
1	$2p^3(1-\text{p}) + 2p^3(1-\text{p}) + 2p(1-p)^3 + 2p(1-p)^3$
4	$p^2(1-p)^2 + p^2(1-p)^2$

From this table, we can compute the mean and variance of $(A-B)^2$:

$$E[(A-B)^2] = -4p^2 + 4p \quad (2)$$

$$Var[(A-B)^2] = 8p^4 - 16p^3 + 4p^2 + 4p$$

Now consider that we have N independent loci. Now, A and B refer to the size-N vector of allele counts in individuals A and B. Because we assume that all loci are independent, simply adding the locus-wise mean and variance for all N loci gives us the mean and variance of the squared Euclidean distance:

$$E[\|A-B\|^2] = \sum_{n=1}^{N} -4p_n^2 + 4p_n$$

$$Var[\|A-B\|^2] = \sum_{n=1}^{N} 8p_n^4 - 16p_n^3 + 4p_n^2 + 4p_n$$

where p_n is the population frequency of the non-reference allele at the nth locus. If N is large, due to the central limit theorem, $\|A-B\|^2$ follows a normal distribution with the mean and variance specified above.

Two individuals and a reference individual.

Now suppose a situation that the distance between A and B cannot be measured, but the distances from A and B to a third person, our reference individual S, are known. This relation is described in the figure below:

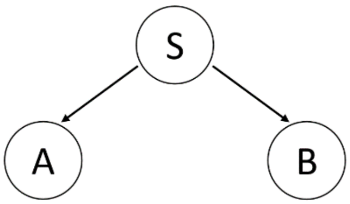

We call this relationship as a triad relationship, where there are two terminal vertices with degree of 1 connected to a single vertex with degree of 2. (Here, degree refers to the number of edges connected to a vertex). In our situation, the distance between each terminal vertex (A or B) and the connection vertex (S) is known. Let $D_{SA} = (S-A)^2$ be the squared Euclidean distance between S and A. Similarly, we define $D_{SB} = (S-B)^2$ and $D_{AB} = (A-B)^2$. We know D_{SA} and D_{SB}, but we do not know D_{AB}.

Our approach is to use $D_{SA} - D_{SB}$ as our measure to check whether A and B are sample overlap. If A and B have the same genetic composition (sample overlap), $D_{SA} - D_{SB}$ will obviously become zero, or close to zero even with genotyping errors. Assume that we only have a single locus. To derive the mean and variance of $D_{SA} - D_{SB}$, we enumerate all possible cases:

S	A	B	D_{SA}	D_{SB}	$D_{SA} - D_{SB}$	Probability
0	0	0	0	0	0	p^6
0	0	1	0	1	-1	$2p^5(1-p)$
0	0	2	0	4	-4	$p^4(1-p)^2$
0	1	0	1	0	1	$2p^5(1-p)$

0	1	1	1	1	0	$4p^4(1-p)^2$
0	1	2	1	4	-3	$2p^3(1-p)^3$
0	2	0	4	0	4	$p^4(1-p)^2$
0	2	1	4	1	3	$2p^3(1-p)^3$
0	2	2	4	4	0	$p^2(1-p)^4$
1	0	0	1	1	0	$2p^5(1-p)$
1	0	1	1	0	1	$4p^4(1-p)^2$
1	0	2	1	1	0	$2p^3(1-p)^3$
1	1	0	0	1	-1	$4p^4(1-p)^2$
1	1	1	0	0	0	$8p^3(1-p)^3$
1	1	2	0	1	-1	$4p^2(1-p)^4$
1	2	0	1	1	0	$2p^3(1-p)^3$
1	2	1	1	0	1	$4p^2(1-p)^4$
1	2	2	1	1	0	$2p(1-p)^5$
2	0	0	4	4	0	$p^4(1-p)^2$
2	0	1	4	1	3	$2p^3(1-p)^3$
2	0	2	4	0	4	$p^2(1-p)^4$
2	1	0	1	4	-3	$2p^3(1-p)^3$
2	1	1	1	1	0	$4p^2(1-p)^4$

2	1	2	1	0	1	$2p(1-p)^5$
2	2	0	0	4	-4	$p^2(1-p)^4$
2	2	1	0	1	-1	$2p(1-p)^5$
2	2	2	0	0	0	$(1-p)^6$

We can summarize the table with respect to $D_{SA} - D_{SB}$,

$D_{SA} - D_{SB}$	Probability
0	$p^6 + 4p^4(1-p)^2 + p^2(1-p)^4 + 2p^5(1-p) + 2p^3(1-p)^3 + 8p^3(1-p)^3 + 2p^3(1-p)^3 + 2p(1-p)^5 + p^4(1-p)^2 + 4p^2(1-p)^4 + (1-p)^6$
1	$2p^5(1-p) + 4p^4(1-p)^2 + 4p^2(1-p)^4 + 2p(1-p)^5$
3	$2p^3(1-p)^3 + 2p^3(1-p)^3$
4	$p^4(1-p)^2 + p^2(1-p)^4$
-1	$2p^5(1-p) + 4p^4(1-p)^2 + 4p^2(1-p)^4 + 2p(1-p)^5$
-3	$2p^3(1-p)^3 + 2p^3(1-p)^3$
-4	$p^4(1-p)^2 + p^2(1-p)^4$

From this table, we obtain

$$E[D_{SA} - D_{SB}] = 0$$
$$Var[D_{SA} - D_{SB}] = 24p^4 - 48p^3 + 20p^2 + 4p$$

Again, consider that we have N independent loci. Then, $D_{AB} = \|A - B\|^2$. Because we assume that all loci are independent, simply adding the locus-wise mean and variance for all N loci gives us the mean and variance of the difference in squared Euclidean distances.

$$E[D_{SA} - D_{SB}] = 0$$
$$Var[D_{SA} - D_{SB}] = \sum_{n=1}^{N} 24p_n^4 - 48p_n^3 + 20p_n^2 + 4p_n \qquad (3)$$

Due to the central limit theorem, if N is large, $D_{SA} - D_{SB}$ will follow a normal distribution with the mean and variance specified above.

Two individuals and multiple reference individuals.

To detect sample overlap, we use a set of reference individuals instead of one. Suppose that we use K references. The relation is described in the figure below:

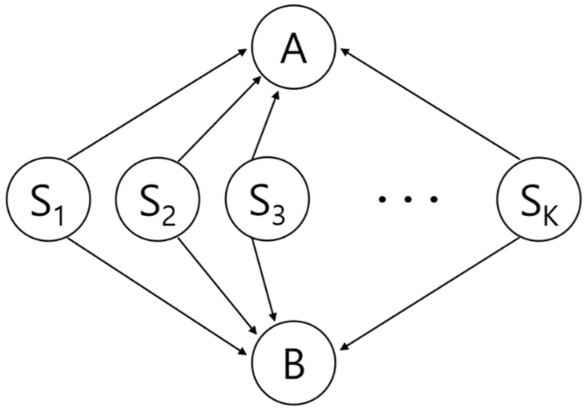

Distances for all edges are known. Thus, we can define distance vectors, referred as V_A and V_B. Distance vector, V_A (or V_B), is a size-K vector in which the ith element contains distance measured between the sample A (or B) to the ith reference individual. That is,

$$V_A = (D_{S_1A}\ , D_{S_2A}\ , \dots , D_{S_KA}) = ((S_1 - A)^2, (S_2 - A)^2, \dots , (S_K - A)^2)$$

$$V_B = (D_{S_1B}\ , D_{S_2B}\ , \dots , D_{S_KB}) = ((S_1 - B)^2, (S_2 - B)^2, \dots , (S_K - B)^2)$$

If A and B are overlapping samples, each element of $V_A - V_B$ will be zero or close to zero even with measuring errors. Thus, we can use the difference of the two distance vectors ($V_A - V_B$) as for our statistic to test sample overlap. To this end, we needed to de-correlate elements of $V_A - V_B$ using covariance matrix. Let Σ be the covariance of $V_A - V_B$ under the null hypothesis that A and B are unrelated. Let $V_A(i)$ be the ith element of V_A. Then we can calculate the (i, j)th element of Σ as follows ($i \neq j$).

$$\Sigma_{ij} = cov(V_A(i) - V_B(i), V_A(j) - V_B(j))$$

$$= cov(V_A(i), V_A(j)) - cov(V_A(i), V_B(j)) - cov(V_B(i), V_A(j)) + cov(V_B(i), V_B(j)) \quad (10)$$

Since D_{S_iA} and D_{S_jB} are uncorrelated, $cov(V_A(i), V_B(j)) = 0$. Thus,

$$= cov(V_A(i), V_A(j)) + cov(V_B(i), V_B(j))$$

Since we assume that A and B are from the same general population,

$$= 2 * cov(V_A(i), V_A(j))$$

$= 2 * cov(D_{S_iA}, D_{S_jA})$

Assume that the reference individuals are from the general population.

Then, $S_i \rightarrow A \leftarrow S_j$ triad relationship is no different from $A \leftarrow S \rightarrow B$ in terms of covariance.

$= 2 * cov(D_{SA}, D_{SB})$

This quantity can be obtained by enumerating all possibilities:

S	A	B	D_{SA}	D_{SB}	$D_{SA}D_{SB}$	Probability
0	0	0	0	0	0	p^6
0	0	1	0	1	0	$2p^5(1-p)$
0	0	2	0	4	0	$p^4(1-p)^2$
0	1	0	1	0	0	$2p^5(1-p)$
0	1	1	1	1	1	$4p^4(1-p)^2$
0	1	2	1	4	4	$2p^3(1-p)^3$
0	2	0	4	0	0	$p^4(1-p)^2$
0	2	1	4	1	4	$2p^3(1-p)^3$
0	2	2	4	4	16	$p^2(1-p)^4$
1	0	0	1	1	1	$2p^5(1-p)$
1	0	1	1	0	0	$4p^4(1-p)^2$
1	0	2	1	1	1	$2p^3(1-p)^3$
1	1	0	0	1	0	$4p^4(1-p)^2$
1	1	1	0	0	0	$8p^3(1-p)^3$

1	1	2	0	1	0	$4p^2(1-p)^4$
1	2	0	1	1	1	$2p^3(1-p)^3$
1	2	1	1	0	0	$4p^2(1-p)^4$
1	2	2	1	1	1	$2p(1-p)^5$
2	0	0	4	4	16	$p^4(1-p)^2$
2	0	1	4	1	4	$2p^3(1-p)^3$
2	0	2	4	0	0	$p^2(1-p)^4$
2	1	0	1	4	4	$2p^3(1-p)^3$
2	1	1	1	1	1	$4p^2(1-p)^4$
2	1	2	1	0	0	$2p(1-p)^5$
2	2	0	0	4	0	$p^2(1-p)^4$
2	2	1	0	1	0	$2p(1-p)^5$
2	2	2	0	0	0	$(1-p)^6$

We can summarize the table with respect to $D_{SA}D_{SB}$,

$D_{SA}D_{SB}$	Probability
0	$p^6 + 2p^5(1-p) + p^4(1-p)^2 + 2p^5(1-p) + p^4(1-p)^2 + 4p^4(1-p)^2 + 4p^4(1-p)^2 + 8p^3(1-p)^3 + 4p^2(1-p)^4 + 4p^2(1-p)^4 + p^2(1-p)^4 + 2p(1-p)^5 + p^2(1-p)^4 + 2p(1-p)^5 + (1-p)^6$
1	$4p^4(1-p)^2 + 2p^5(1-p) + 2p^3(1-p)^3 + 2p^3(1-p)^3 + 2p(1-p)^5 + 4p^2(1-p)^4$
4	$2p^3(1-p)^3 + 2p^3(1-p)^3 + 2p^3(1-p)^3 + 2p^3(1-p)^3$
16	$p^4(1-p)^2 + p^2(1-p)^4$

From this table, we obtain

$$E[D_{SA}D_{SB}] = 12p^4 - 24p^3 + 10p^2 + 2p$$

Since we know $E[D_{SA}] = E[D_{SB}] = -4p^2 + 4p$ from Eq (2),

$$cov(D_{SA}, D_{SB}) = E[D_{SA}D_{SB}] - E[D_{SA}]E[D_{SB}]$$

$$= 12p^4 - 24p^3 + 10p^2 + 2p - (-4p^2 + 4p)^2$$

$$= -4p^4 + 8p^3 - 6p^2 + 2p$$

Again, consider that we have N independent loci. Let A, B, and S refer to the size-N vector of allele counts in individuals A, B, and S. Because we assume that all loci are independent, simply adding the locus-wise covariance for all N loci gives us the covariance in a triad relationship.

$$cov(D_{SA}, D_{SB}) = \sum_{n=1}^{N} -4p_n^4 + 8p_n^3 - 6p_n^2 + 2p_n$$

Therefore, we have

$$\Sigma_{ij} = 2 * cov(D_{SA}, D_{SB})$$

$$= \sum_{n=1}^{N} 2(-4p_n^4 + 8p_n^3 - 6p_n^2 + 2p_n)$$

$$= \sum_{n=1}^{N} -8p_n^4 + 16p_n^3 - 12p_n^2 + 4p_n \qquad (4)$$

Since the diagonal elements of Σ are specified by Eq (3) and non-diagonal elements are specified by Eq (4), we completely specified Σ.

2.3 P-value

Our null hypothesis is that the two individuals are unrelated and our alternative hypothesis is that the two individuals are a sample overlap. Our statistic s_{overlap} in Eq (1) has a property that, under the alternative hypothesis, it comes close to zero rather than going away from zero. Thus, the P-value can be calculated by the lower tail (not the upper tail) volume of the K df χ^2 distribution.

2.4 Significance threshold

There are two ways to set the significance threshold for our sample overlap test. The first is to consider repeated comparisons to minimize family-wise error rate. For example, if we test every possible pair of 1,000 samples, the threshold will be $\alpha = 0.05/\binom{1000}{2} \approx 1 \times 10^{-7}$ by the Bonferroni correction. Another way is to let our method to choose an appropriate threshold. As shown in **Fig. 1f**, our statistic s_{overlap} forms distinctive clusters for unrelated individuals and overlapping samples. A threshold corresponding to the middle value of these two clusters can provide an appropriate balance between specificity and sensitivity. However, this value will depend on the specific set of SNPs used for the analysis, their allele frequencies, and the reference individual dataset. Our software implementation includes a module that, given all this information, simulates a large number of pairs of unrelated and overlapping samples, figures

out the mean and variance of the statistic for the two clusters, and chooses an appropriate middle value automatically.

2.5 False positive rate and power simulation

To check the false positive rate, we simulated random pairs of individuals under the null hypothesis that the two individuals are unrelated. We assumed 1,000 loci (N=1,000). For each locus i, we randomly chose the reference allele frequency p_i from the uniform distribution in the range $(0.05, 0.95)$. Given p_i, each simulated individual was assigned an allele count $g \in \{0, 1, 2\}$ drawn from $Binom(2, p_i)$. We simulated a pair of unrelated individuals and calculated the statistic s_{overlap} using 20 reference individuals (K=20) to obtain P-value. The 20 reference individuals were newly generated for each pair to test so that we can avoid bias due to using a specific reference dataset. We tested 1,000,000 pairs of individuals to check the false positive rates, assuming 1% genotyping error rate. **Fig. S2** shows that the empirical null distribution of our test statistic well matched to the asymptotic χ^2 distribution (20 df). We estimated the false positive rate at a given significance threshold α as the proportion of simulated pairs with P-value $\leq \alpha$. The false positive rate was well controlled at varying thresholds from 0.05 to 0.0001 (**Table S1**).

In order to use the asymptotic distribution for P-value calculation, we will need a sufficiently large N. We found that we need a large N (>100) as well as a large ratio of N/K (>20) for accurate approximations (**Fig. S8**). Fortunately, these requirements are easy to meet in practical situations. Most of the currently available genotyping platforms provide >1,000 independent loci after LD pruning, thereby satisfying N>100 and N/K>20 requirements easily if we assume we use several tens of reference individuals (K).

To check the power, we simulated the alternative hypothesis that the pair of individuals was a sample overlap. We assumed a genotyping error rate of 1%. While varying the number of loci (N) and the number of reference individuals (K), we checked the power using 100,000 simulated pairs of individuals. We used the significance threshold $\alpha = 10^{-7}$. The power at each set of N and K is shown in **Fig. 1e**. The result showed that power is dependent on both N and K, but the major determinant is K.

Based on our power and false positive rate results, we chose to use N=1,000 and K=30 (or N=500 and K=20) in various simulations below. The reasons for this choice are the following. (1) We have >20 reference individuals in each population of the HapMap or the 1000Genomes data. (2) We will need a ratio of N>K×20 for an appropriate control of false positive rate. (3) Most of genotyping platforms will provide >1,000 independent SNPs after pruning. Note that in many real situations, we can increase N and K larger than these choices, which will increase power as shown in **Fig. 1e**.

2.6 Real data simulation using WTCCC

To simulate sample overlap detection with real data, we used 1,963 samples of Type 1 diabetes(T1D) cases and 1,480 samples of the 1958 British Birth Cohort(58C) controls in the Wellcome Trust Case Control Consortium (WTCCC) data [3]. We split T1D cases and 58C controls into 3 disjoint case/control studies. Then we randomly chose individuals and added them to the other studies, such that each pair of studies would have 10∼30 overlapping samples. In the end, Study A(# of sample=1,180) and Study B(# of sample=1,158) overlapped in 25 samples, Study A(1,180) and Study C(1,158) overlapped in 13 samples,

and Study B(1,158) and Study C(1,158) overlapped in 13 samples. In total, the 3 studies included 3,496 samples, when double-counting overlapping samples. The design of 3 studies is summarized in the figure below.

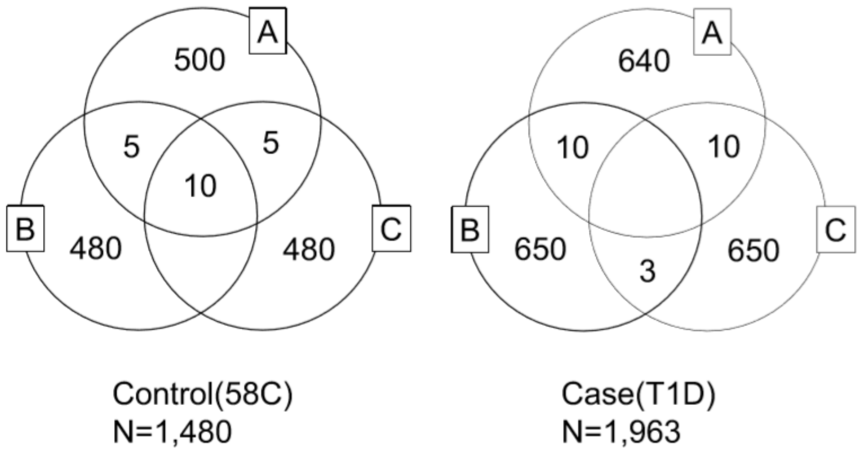

We utilized 50 randomly selected samples from the 1000Genomes data as our reference set (see "Section 6. Datasets used in the Analysis"). We pruned 1000Genomes SNPs based on physical distance (retaining those > 5Mb) and applied quality control (call rate > 0.95) to obtain 161,235 independent SNPs. Given 7,790 overlapping SNPs between the WTCCC data and the 1000Genomes pruned independent SNPs data. Missing alleles were imputed with random selection from {0,1,2}. There were 4,073,844 pairs of individuals across three groups, as shown in the figure below.

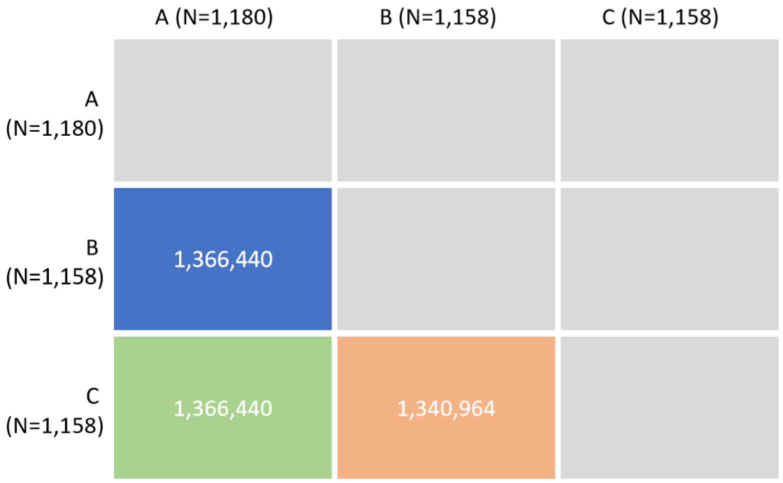

Here, we used the significance threshold 1.2×10^{-8} ($\approx 0.05/4{,}073{,}844$). Applying our method gave us perfect sensitivity (100%) and specificity (100%) for finding overlapping pairs (**Fig. 1f**).

Although we selected 50 overlapping samples randomly for this analysis, we wanted to exclude the possibility that our method worked well because of some characteristics that these samples happened to have. Therefore, we performed additional analysis. We sampled 1,000 unrelated pairs and 1,000 overlapping samples from the WTCCC dataset. We used the same 1,000Genomes data as reference and

used the same threshold. In this additional analysis, the distinction by our method was still perfect, showing that the results were not affected by sampling bias (**Fig. S9**).

2.7 Identification of relatives

In terms of genetic contents, overlapping samples are no different from twins. If the tested pairs are close relatives, it is possible that our statistic s_{overlap} in Eq (1) can have a smaller expected value than unrelated pairs. In that sense, our method may possibly be used to distinguish relatives. We performed simulations to examine if our statistic can distinguish relatives, and if yes, to what extent. To this end, we simulated pairs of individuals that are relatives of different degrees. We gradually increased the degree from twins (equivalent to overlapping samples) to 1st degree, 2nd degree, 3rd, and unrelated. For each degree, we simulated 100,000 pairs. For each pair, the genotype dosage of each SNPs was simulated from a binomial distribution g~$Binom(2, p_i)$, p_i~$Unif(0.05, 0.95)$, and the randomly selected SNPs proportional to their coefficient of relationship were set to have the same genotype. We used N=1,000 and K=30 for this simulation. Other settings were similar to our false positive rate simulation.

Density plot of our statistic under different degrees of relationships is shown in **Fig. S4a**. As described previously, the two clusters for twin pairs and unrelated pairs showed clear distinction. The 1st degree relatives formed a cluster that was located between the two clusters. This cluster was clearly distinctive from the twins but had some overlap with unrelated pairs. We calculated the posterior probability of relationship as the density of the distribution of a specific relationship divided by the sum of densities of all relationships, assuming a uniform prior (**Fig. S4b**). Then we predicted the relationship of each pair as the relationship with the highest posterior probability. Given the true relationship being the 1st degree, 79% of simulated pairs were predicted to be the 1st degree (**Fig. S4c**). Thus, we can say that the 1st degree relatives were generally distinguishable, although the distinction may not be perfect in some cases. In contrast, starting from the 2nd degree relatives, the cluster had much larger overlap with unrelated pairs. For example, given the 2nd degree relatives, only 39% of simulated pairs were predicted to be the 2nd degree. Next, we measured how precisely our predicted relationship represents true relationship. Out of all pairs predicted to be each degree of relationships, we measured the proportion of the correct prediction (thus, "precision"). The precisions were 100% for sample overlap, 65% for 1st degree relatives, 36% for 2nd degree relatives, 35% for 3rd degree relatives, and 51% for unrelated pairs (**Fig. S5)**. Thus, we can say that for the 2nd or higher degree relationships, our method may not have sufficient distinctive power to predict the relationship correctly. The detailed result of prediction performance is described in **Fig. S5**.

2.8 Genetic relatedness metric

Although we kept using the squared Euclidean distance as our metric of genetic distance for sample overlap detection, the genetic relatedness can also be used. The genetic relatedness is a commonly used measure of genetic distance in quantitative genetics, for example for calculating the genetic relationship matrices (GRMs) in heritability estimation [4]. Let $X_{t,n} \in \{0,1,2\}$ be the reference allele count of individual t at SNP n. Let p_i be the reference allele frequency of locus i, which can be obtained from independent reference data or the sample data itself. Let $p_n = (\sum_{b=1}^{B} X_{b,n} + 1)/(2\text{B} + 2)$ [5], where B is

the number of data used. We then standardize $X_{t,n}$ such that $\bar{X}_{t,n} = (X_{t,n} - 2p_n)/\sqrt{2p_n(1-p_n)}$ [4]. The genetic relatedness between individuals t and u is defined as,

$$G_{t,u} = \frac{1}{N}\sum_{n=1}^{N} \bar{X}_{t,n}\bar{X}_{u,n} \text{ [4]}.$$

If we use the genetic relatedness as our metric, we need a different way of obtaining the covariance matrix Σ in Eq (1), because it is not easy to analytically derive Σ as we have done in Eq (3) and (4) for the squared Euclidean distance metric. Instead, we can empirically estimate Σ. Given the set of SNPs and their allele frequencies, it is possible to generate a large number of unrelated individuals, calculate the difference in distance vectors ($v_t - v_u$ in Eq (1)), and estimate the covariance.

We simulated 100,000 random pairs and calculated distance vectors to a fixed set of 20 references using 500 loci (K=20 and N=500). We estimated the covariance matrix from the 100,000 vectors of $(v_t - v_u)$. We then used this estimated covariance in Eq (1). We examined if the use of estimated covariance works well by simulating data under the null and alternative hypotheses. We simulated 10,000 pairs under the null hypothesis (pairs are unrelated) and 10,000 pairs under the alternative hypothesis (pairs are sample overlaps). The P-values under the null hypothesis were uniformly distributed (**Fig. S3a**). Also, the sample overlap formed a clearly distinguishable cluster in the histogram of statistics (**Fig. S3b**). These results showed that the genetic relatedness can effectively be used for sample overlap detection by using the empirically estimated covariance matrix.

3 Principal component map construction

3.1 Method

Distance vectors can be used for several population genomic analyses. First, they can be used for constructing the spatial structure in the principal component (PC) map. As Novembre et al. [6] showed, two-dimensional PC plot can approximate the geographical origins of individuals. Typically, actual genotype data of individuals are required to calculate PCs. We developed a method to approximate the PC map using only distance vectors without requiring actual genotype data.

The core idea of our approach is to estimate the location of our samples on the PC map of reference data. The procedure begins with generating the PC map of the reference data first. The reference individuals in this map serve as “fixed anchors”. Then, we put each target individual onto this map using a distance vector. Finally, we erase the reference individuals, which leaves us the PC map of target individuals.

Consider that we have K reference individuals. We first approximate the coordinates of the K references by applying principal component analysis (PCA) to their GRM. The top two eigenvectors (PCs) from the PCA can be plotted in a two-dimensional (2D) space, $\mathcal{P}$. For each target individual, we approximate the position of the individual in $\mathcal{P}$ as follows. First, we calculate the target’s distance vector to K references based on the genetic relatedness metric. For this application, the use of genetic relatedness metric is natural because PCA is closely related to GRM. Then, we construct the GRM for the K+1 individuals (the reference set and the target) by appending the target’s distance vector to the rows and columns of the GRM of the references. We apply PCA to this size-$(K+1)\times(K+1)$ GRM to obtain a new principal component map of K+1 individuals in a new 2D space, $\mathcal{P}'$. The positions of the K references in $\mathcal{P}'$ are similar to their positions in $\mathcal{P}$ (after adjusting for rotation); however, they are not identical, because adding one more datapoint in the PCA can distort the positions of the other points (**Fig. S6**).

Because of this subtle difference between $\mathcal{P}$ and $\mathcal{P}'$, although we know the target sample's location in $\mathcal{P}'$, we need a procedure to project that point from $\mathcal{P}'$ to $\mathcal{P}$. Interestingly, we can apply another layer of "multilateration technique" to overcome this slight difference between $\mathcal{P}$ and $\mathcal{P}'$. Using the map in $\mathcal{P}'$, the 2-D Euclidean distances between the target and the references can be calculated to create a distance vector. (Note that this distance vector is not for the genetic distance used for GRM, but for the 2-D distance on the PC map). Using the standard multilateration technique, this distance vector can be used to map the approximate position of the target on $\mathcal{P}$. In particular, the least-square minimization method [7] is used as follows. Let (x, y) be the unknown position of the target individual in $\mathcal{P}$. Let (x_k, y_k) be the coordinate of the kth reference in $\mathcal{P}$. Let r_k be the distance between the sample and the kth reference calculated in $\mathcal{P}'$. Let $\hat{r}_k$ be the distance between the sample and the kth reference in $\mathcal{P}$. We minimize the function

$$F(x,y) = \sum_{k=1}^{K} (\hat{r}_k - r_k)^2 = \sum_{k=1}^{K} \left(\sqrt{(x-x_k)^2 + (y-y_k)^2} - r_k\right)^2$$

to approximate (x, y) in $\mathcal{P}$. This procedure is repeated for each target individual. After all repetitions, the approximated PC map of target individuals is obtained by removing reference data from the plot.

3.2 Real data analysis using POPRES

We performed real data analysis using POPRES data [8]. See "Section 6. Datasets used in the analysis" for details of the dataset. We used 1,387 samples from the European ancestry.

POPRES as a reference set

The first analysis was to use POPRES for both target samples and reference. We subsampled 40% from each population of POPRES data and used them as our reference set. The rest (60%) of the data was used as our target samples. This splitting gave us 572 reference individuals and 815 target samples. Missing genotypes were assigned with value $2p_i$, where p_i was the allele frequency inferred from the reference individuals. A total of 197,146 variants were used to calculate genetic relatedness between any two individuals. For this application, we did not prune SNPs, similar to Novembre et al. [6].

As described in our main manuscript, the approximated PC map based on distance vectors (**Fig. 2a**) greatly resembled the original PC map based on actual genotypes (**Fig. 2b**). The samples from same geographic regions were clustered together and the populations were distinguishable. Populations geographically adjacent were found near each other and populations geographically apart were found far from each other. Also, some populations in our result corresponded to the geographic outline of Europe. Spanish and Portuguese samples (population codes: SP and PT in **Fig. 2a**) formed a shape similar to Iberian Peninsula, Italian samples (population code: IT in **Fig. 2a**) formed a shape similar to Italian peninsula, and English and Irish samples (population codes: UK and IE in **Fig. 2a**) formed a shape similar to two main islands of United Kingdom.

The accuracy of the spatial structure mapping can depend on the number of variants used. We gradually decreased the number of SNPs used in this analysis by subsampling SNPs. As expected, the resolution of mapping was reduced as fewer SNPs were used (**Fig. S10**). In particular, the resolution drastically decreased when the number of SNPs was reduced from 50,000 to 10,000.

1000Genomes as a reference set

Then, we used samples from the 1000Genomes data [2] as our reference. See "Section 6. Datasets used in the analysis" for details of the dataset. Out of 1,092 samples in the 1000Genomes phase I dataset, we selected 305 European samples from populations TSI, GBR, and IBS. We excluded CEU because the population was from USA, and FIN which was underrepresented in POPRES. We used all 1,387 POPRES samples as our target samples. Because the reference and target samples were independent datasets, we screened for variants shared between the two datasets and matched strands and reference alleles. A total of 196,350 shared variants were used to calculate genetic relatedness between any two individuals.

Fig. S7 shows that the constructed PC map based on distance vectors, which roughly resembled the European map. However, the resolution decreased when compared to the PC map based on genotype data (**Fig. 1a** of Novembre et al. [6]) or when compared to our analysis using subsamples of POPRES as reference (**Fig. 2a**). For example, it was hard to distinguish Eastern Europe and Russian populations, where they were entangled with central Europe population. The lower resolution was expected, because our method uses the PC map of reference data as "anchors" and therefore depends on how much variability of the target samples the reference data represents. Indeed, the 1000Genomes data lacked references from Eastern Europe and Russia [2]. We expect that the resolution of this analysis will keep increasing as more diverse and ample reference datasets are built.

Map rotation

For this real data analysis, we rotated the maps to facilitate the visual comparison to the physical map of the Europe. For this purpose, we used the following method described in Novembre et al. [6]. We searched for the rotation angle θ which maximizes the function:

$$f(\theta) = Cor(lat, x'(\theta, v_1, v_2)) + Cor(long, y'(\theta, v_1, v_2))$$

where Cor is the correlation function, lat and $long$ are the vectors of the latitude and longitude of each reference according to their geographic origin, $x'$and y' are functions for 2D rotation about a point, and v_1 and v_2 are the approximated coordinates of the samples. Specifically, when a point located at (x, y) is rotated about the origin with a rotation angle of θ, the new location of the point (x', y') will be

$$x' = xcos\theta - ysin\theta$$

$$y' = ycos\theta + xsin\theta$$

4 Ancestry estimation

4.1 Method

Another population genomic analysis application that distance vectors can be used for is ancestry estimation. We developed a method to use distance vectors to infer the ancestry composition of an individual. Our method works in a supervised way, requiring the reference data of candidate populations. The method is built upon the PC map construction described in section 3. The idea is to approximate the location of a target individual in the PC map of the reference populations. Then, we measure the Euclidean distance of the individual to the centroid of each population in the PC map. The ancestry

proportion is estimated as being inversely proportional to these distances. For example, if we use the CEU and YRI of 1000Genomes as our reference, and the distance to each population was 10 and 5, then the ancestry proportion is estimated as 1/10:1/5=1:2. If we use two candidate populations, we use the 1 dimensional Euclidean distance (difference in PC1). If we use more than two candidate populations, we use the 2 dimensional Euclidean distance in the PC1-PC2 map. Note that there exist other methods that can assign individuals to population groups or decompose the ethnic composition of an individual, such as STRUCTURE [9] or ADMIXTURE [10]. These methods require genotype data of individuals and can run in an unsupervised way without the need for training or reference data.

4.2 Two population simulation

To benchmark our method, we simulated admixed individuals from two populations. We used the Japanese (JPT) and the British (GBR) population data from the 1000Genomes data. To generate haplotypes of admixed populations, we used the Hapgen2 software [11]. We randomly chose 50 samples from each of the two populations and used them as the ancestry pool data for Hapgen2. The rest of the population data was used as the reference to calculate distances to. Using Hapgen2, we generated samples with different ratios of mixture (1:9, 3:7, 5:5, 7:3, 9:1). Then, we used our method to estimate the admixture proportion. As shown in **Fig. 2c**, the estimation of the proportion was highly accurate (r^2=0.98). **Fig. S11a** shows the samples in the 2D PC map.

We then tried a harder task; we admixed two European populations, British (GBR) and Toscani in Italia (TSI). Since these two populations are genetically close, we can expect that decomposing the proportion will be more difficult. Using Hapgen2, we similarly constructed samples of different ratios of mixture. As shown in **Fig. 2c**, although the estimation was not as accurate as the previous simulation, the estimated and true proportions showed positive correlations (r^2=0.86). **Fig. S11b** shows the samples in the 2D PC map.

4.3 Three population simulation

Here we simulated admixed individuals from three populations. Similar to previous simulation, we used Hapgen2 to simulate admixed individuals. We used CHS, GBR, and YRI from the 1000Genomes data and assumed varying proportions of the three populations. For this dataset, we also ran ADMIXTURE for comparison. ADMIXTURE is different from our method in that it requires genotype data and does not require the reference data. We used genotype data for ADMIXTURE and ran ADMIXTURE in a semi-supervised fashion by giving the correct number of populations as input (K=3) and running the reference individuals together with the target individuals. This way, the reference individuals from the three populations form three distinct cluster in the ADMIXTURE algorithm so that the target individual can be proportionally assigned to each cluster. Again, to avoid data re-use, we used 50 samples from each reference population (CHS, GBR, and YRI) for data generation using Hapgen2 and used the rest as the reference data for our method or for ADMIXTURE.

We compared the estimated proportions of our method and of ADMIXTURE. Our method and ADMIXTURE gave similar output and the estimated proportions were similar to the true proportions (**Fig. 2d**). **Fig. S12** shows the samples in the 2D PC map.

5 Unidentifiability

Because our method conceals the genotype data of an individual and only shares a distance vector to reference data, it is important that the genotype data should not be recovered by the information in the distance vector. That is, we don't want the exact position of the individual in the N-dimensional space to be identified or specified by the distance vector. We describe mathematical proofs and simulations related to this issue. Note that we use the term "unidentifiability" to describe the condition that the actual genotypes cannot be recovered, but not the condition that the individual identity cannot be identified. The latter can have different meanings depending on the context; if one has distance vectors of a group of individuals and wants to identify if a new individual is in the group, it is certainly possible by comparing distance vectors as described in our application of overlapping sample detection.

5.1 Mathematical proofs

We present several mathematical proofs related to this issue. These proofs make a general assumption that the coordinates of a point are real-valued. This assumption is appropriate for typical situations that multilateration is applied (e.g. aircraft navigation).

Condition under which point is identifiable

We prove that under certain conditions, the target point can be uniquely determined. Let $p_0, \dots, p_k$ be $k+1$ distinct points in the n-dimensional Euclidean space $\mathbb{R}^n$. Assume a point $x \in \mathbb{R}^n$ whose precise location is not known but the distance $r_i := d(x, p_i)$ for each i is known. Note that if $r_i + r_j < d(p_i, p_j)$ for some $i \neq j$, then no x satisfies the given data. Also if $r_i + r_j = d(p_i, p_j)$ for some $i \neq j$, then the possible position of x is already uniquely determined by only p_i, p_j. This means that the interesting case to analyze is when $r_i + r_j > d(p_i, p_j)$ for all $i \neq j$. Therefore, we suppose the following assumption.

$$r_i + r_j > d(p_i, p_j), \forall i \neq j.$$

An *affine hyperplane* of $\mathbb{R}^n$ is an $(n-1)$-dimensional affine subspace of co-dimension 1. In other words, one can think of a linear subspace of $\mathbb{R}^n$ of dimension $n-1$ and take a parallel copy by translating the subspace along the normal direction. An $(n-1)$-dimensional space obtained this way, we call it affine hyperplane or shortly just hyperplane (since this is the only type of hyperplane we will consider here).

For $p \in \mathbb{R}^n$ and $R > 0$, let $S_R(p)$ denote the set of points in $\mathbb{R}^n$ whose distance from p is precisely R. It is clear that $S_R(p)$ is a $(n-1)$-dimensional sphere with radius R and center p.

For each i, let S_i denote the sphere $S_{r_i}(p_i)$. By definition, x lies in the intersection of these sets S_i, i.e., $x \in \bigcap_{i=0}^{n} S_i$. Note that for any $i \neq j$, S_i is different from S_j. In a special case when S_i and S_j are tangent to each other, i.e, the intersection is just one point, x is already uniquely determined. Hence, we assume it is not the case. In this case, $S_i \cap S_j$ lies on the unique hyperplane $H_{i,j}$.

Now we prove the following claim:

> ***Proposition 1.*** *If $k = n$ and $p_0, \dots, p_n$ are affinely independent (i.e., $p_1 - p_0, p_2 - p_0, \dots, p_n - p_0$ are linearly independent), then the position of x is uniquely determined.*

Proof.

For each $i = 0, \ldots, n$, say H_i is the unique hyperplane containing the intersection between S_0 and S_i. It is clear that the vector $u_i := p_i - p_0$ is a normal vector to H_i. Since $x \in \cap_{i=0}^{n} S_i$, we also have $x \in \cap_{i=1}^{n} H_i$. Now it suffices to show that $\cap_{i=1}^{n} H_i$ has dimension 0, i.e., just a single point.

We first consider the following general lemma.

Lemma 1. *Suppose that there are m hyperplanes in $\mathbb{R}^n$ whose normal vectors are v_i and their intersection is nonempty. Then the intersection of those hyperplanes has dimension of at least $n - m$, and exactly $n - m$ when v_i are linearly independent.*

Let $W_1, \ldots, W_m$ be affine subspaces in $\mathbb{R}^n$ where $\dim W_i = d_i$, and $\dim(W_1 \cap \ldots \cap W_\ell) = i_\ell$. Also let $W = W_1 \cap \ldots \cap W_m$. Since we are assuming W is nonempty, $W_1 \cap \ldots \cap W_\ell$ is nonempty for all ℓ.

Note that $\dim S + \dim T - \dim(S \cap T) \leq n$ for any affine subspaces S and T of $\mathbb{R}^n$. Applying this to W_1 and W_2, we have $d_1 + d_2 - i_2 \leq n$, hence $i_2 \geq d_1 + d_2 - n$. Applying this again to $W_1 \cap W_2$ and W_3, one gets $i_2 + d_3 - i_3 \leq n$, i.e., $i_3 \geq i_2 + d_3 - n \geq d_1 + d_2 + d_3 - 2n$. By induction, one can easily show that $\dim W = i_m \geq d_1 + \cdots + d_m - (m-1)n$.

Because W_i are hyperplanes, $\dim W_i = n - 1$ for all i. Hence, $\dim W \geq m(n-1) - (m-1)n = n - m$. Thus, we proved that the intersection has dimension of at least $n - m$.

Now let v_i be a normal vector to W_i for each i, and assume that they are linearly independent. For any vector w contained in W, w is orthogonal to each v_i. Thus, $v_1, \ldots, v_m$ are independent vectors in $W^\perp$, which gives us $\dim W + m \leq n$. Since we already have the inequality in the other direction, this implies that $\dim W$ is precisely $n - m$. This completes the proof of *Lemma 1.* □

Now, let's come back to the proposition. Since $u_1, \ldots, u_n$ are linearly independent, $\dim H$ is precisely $n - n = 0$ when $H = \cap_{i=1}^{n} H_i$. Hence, it is just a single point. This proves *Proposition 1.* □

Summary: This proof shows that, in an n-dimensional space, if we have $n+1$ reference datapoints to measure distances to, the target point can be exactly specified in general. For example, in the 3-dimensional space, we will need 4 satellites to specify the location of an aircraft. (In practice, 3 satellites can suffice because of the additional condition that the aircraft has a lower altitude than satellites.)

Condition under which point is unidentifiable

We prove that under certain conditions, the target point cannot be uniquely determined. We argue that if $n \geq k + 1$, the exact location of x cannot be identified.

Since $x \in S := \cap_{i=0}^{k} S_i$, we want to obtain the lower bound of *dimS*. Given $k+1$ points in $\mathbb{R}^n$, we can consider every possible pair of points. Each pair of $k+1$ points gives us a hyperplane where x must belong. Imagine that we have an intersection H' of all $\binom{k+1}{2}$ hyperplanes. $S = \cap_{i=0}^{k} S_i$ will be a sphere that resides in H' and will have one less dimension than H'. This remark about the dimension of S compared with the dimension of H' will be justfied after we first explain how to understand H' below.

First of all, what is the dimension of H'? It turns out that to obtain the intersection H', it suffices to consider only k hyperplanes. Write our $k+1$ points as $p_0, \ldots, p_k$ and define $u_i = p_i - p_0$ for each i as before. Then the hyperplane determined by p_i *and* p_j has $p_i - p_j$ as a normal vector, but this vector is already in the span of u_i and u_j. This means when we intersect $H_i \cap H_j$ with the hyperplane plane determined by p_i and p_j, the dimension does not go down, i.e., $H_i \cap H_j$ is contained in the hyperplane determined by p_i and p_j. By definition, $H_i \cap H_j$ is precisely the set of vectors which is orthogonal to both u_i and u_j, hence they are already orthogonal to $u_i - u_j$. From this, we conclude that the intersection of $\binom{k+1}{2}$ hyperplanes obtained from the $k+1$ given points is the same as the intersection of H_i's we considered in the proof of the *Proposition 1*.

Now note that by definition, H_1 is the smallest affine subspace containing the sphere $S_0 \cap S_1$, hence the dimension of $S_0 \cap S_1$ is one lower than the dimension of H_1. Recall that we are working on the mild assumption that $r_i + r_j > d(p_i, p_j), \forall i \neq j$. This is equivalent to saying that the sphere S_i and S_j intersect non-tangentially (or, transversally). In practice, it is also safe to assume further that the spheres we get as the intersection of S_i's also intersect transversally. Not only such a condition is satisfied with probability 1, also it is always guaranteed if we perturb our data by introducing an error term as in the next section (lattice case). Under this assumption, the spheres $S_0 \cap S_1$ and $S_0 \cap S_2$ intersect transversally, hence $H_1 \cap H_2$ is the smallest affine subspace containing the sphere $(S_0 \cap S_1) \cap (S_0 \cap S_2) = S_0 \cap S_1 \cap S_2$. By induction, we conclude that $\cap_{i=1}^k H_i$ is the smallest affine subspace containing $\cap_{i=0}^k S_i$. We just showed above that $H' = \cap_{i=1}^k H_i$, hence we know that $\cap_{i=0}^k S_i$ has one less dimension than H'.

Recall that by Lemma 1 the intersection of all H_i's has dimension of at least $n-k$ provided that $n-k \geq 0$ and the intersection is nonempty (which follows from our assumption that x exists). Note that $x \in \cap_{i=0}^k S_i \subset \cap_{i=1}^k H_i$. Since $\cap_{i=0}^k S_i$ is a $(n-k-1)$-dimensional sphere in the affine space $\cap_{i=1}^k H_i$ of dimension $n-k$, as long as $n-k \geq 1$, the set of possible locations of x is more than one point. Hence, the final conclusion is that if $n \geq k+1$, either x cannot exist or x exists but its location cannot be uniquely determined under the assumption that $r_i + r_j > d(p_i, p_j)$ for all $i \neq j$.

Lattice case

The proofs above depend on the assumption that the coordinates are real-valued. If the points are in a more constrained space, the conditions for identifiability and unidentifiability may change. Here, we consider a lattice space (e.g. all integers). Assume that our data set $\{p_0, \ldots, p_k, x\}$ is contained in some lattice L in $\mathbb{R}^n$. Let H be as in the previous section. As we saw before, $dim H \geq n-k$. Hence, if $n-k > 0$, H itself does not determine the exact location of x.

The set of possible locations of x is now $H \cap L$. So, as long as $n-k < n$, i.e., $k > 0$, H may intersect L only at one point. This causes a problem for our method, since when $H \cap L$ is a single point, the exact location of x is completely determined, and this phenomenon happens very often. In all practical purposes, it is enough to assume that L is the integral lattice $\mathbb{Z}^n$. We will assume this for the rest of this section.

Mathematically speaking, a generic (randomly chosen) affine subspace will miss the lattice L. As an instructive example, we describe the situation in dimension 2, i.e, let's consider $\mathbb{R}^2$ with the integral lattice $\mathbb{Z}^2$. For a straight line in $\mathbb{R}^2$ to intersect more than one point in $\mathbb{Z}^2$, it must have a rational slope. On the other hand, the set $\mathbb{Q}$ of rational numbers has Lebesgue measure zero as a subset of $\mathbb{R}$ (said differently, a

randomly chosen real number is irrational with probability one). Hence, a random straight line would intersect $\mathbb{Z}^2$ at most one point.

Here is our suggestive solution for the above problem; instead of using $r_i = d(x, p_i)$, we perturb the given data by introducing an error term. More precisely, choose a small positive number ϵ and define $d_{i'} = r_i + \epsilon_i$ where ϵ_i is a number chosen randomly in the interval $(-\epsilon, \epsilon)$. The actual values of r_i and ϵ_i are hidden and only the value of $d_{i'}$ is given to the user of the data set. Then H_i is replaced by the ϵ_i-neighborhood of H_i, and at the end H is replaced by μ-neighborhood of H, call it H_μ where $\mu = \min\{\epsilon_i\}$. If one can show $H_\mu \cap L$ contains infinitely many points, then we can overcome the problem we described above.

Obviously, if μ is arbitrarily small, this still does not hold. Fortunately, the following mathematical statement is true for an obvious reason. There exists $\epsilon_0 > 0$ such that as long as $\mu \geq \epsilon_0$, then $H_\mu \cap L$ contains infinitely many points. Hence, one can take a positive number ϵ bigger than ϵ_0, and choose each ϵ_i in the set $(-\epsilon, -\epsilon_0) \cup (\epsilon_0, \epsilon)$. For instance, in $\mathbb{R}^n$, it would be enough to take ϵ_0 to be $\sqrt{N}$. But this choice of ϵ_0 is quite large, and one might want to choose ϵ_0 as small as possible. While we do not fully resolve this optimization problem, we note that a very famous theorem of Hurwitz [12] implies that one can take ϵ_0 to be $1/\sqrt{5}$ in dimension 2, so it is plausible that one might be able to take a quite small number as ϵ_0 in more general case.

Now we explain the situation in dimension 2 with more details. Say we choose a line with irrational slope r. Without loss of generality, let's assume this line actually passes through the origin and call it P. Then P is the set of solutions of the equation $y = rx$. If $(a, b) \in \mathbb{Z}^2$ is contained in the region B bounded by two lines $y = rx + \epsilon$ and $y = rx - \epsilon$, then surely (a, b) lies in the ϵ −neighborhood of P. On the other hand, $(a, b) \in B$ is equivalent to $|ar - b| < \epsilon$. Alternatively, one can write $|r - \frac{b}{a}| < \frac{\epsilon}{a}$. Hurwitz's theorem says $|r - \frac{b}{a}| < \frac{1}{\sqrt{5}a^2}$ is satisfied by infinitely many $(a, b) \in \mathbb{Z}^2$. In particular, $(1/\sqrt{5})$-neighborhood of P intersects $\mathbb{Z}^2$ at infinitely many points. Hence, in dimension 2, it is enough to take ϵ_0 to be $1/\sqrt{5}$.

5.2 Simulations

Although we provided a mathematical proof about the unidentifiability condition, that only applies to the spaces of real values. The lattice space case is not directly relevant to our context, because the allele count in genome data is in a highly constrained integer space, $\{0,1,2\}^n$. Therefore, we performed extensive simulations to show that in practice, the original genome data is not recoverable given distance vector information.

To this end, we designed a greedy algorithm that aims to recover the original genome.

Algorithm 1: Greedy algorithm

INPUT: Reference data R, distance vector v, and allele frequencies of the SNPs $\{p_i\}$

OUTPUT: A prediction for target sample t

REPEAT 1,000 times

Randomly generate a sample t' based on $\{p_i\}$

Calculate distance vector v' between t' and R

Calculate sum of squared error (SSE) between v' and v

WHILE True:

Randomly select a SNP j

Try changing SNP j to two alternating forms (e.g. $0 \rightarrow 1$, $0 \rightarrow 2$)

If an alternating form reduces SSE, accept the change

If no change reduces SSE for a long period (1,000 trials), break loop

Record solution t'

END REPEAT

Choose the best solution with the minimum SSE among 1,000 repeats.

Starting from the random genotypes, the algorithm stepwisely moves the space to find the solution whose distance vector most resembles the input distance vector. The algorithm quits if no improvement is obtained for a prolonged time. To avoid local optimum, the algorithm restarts with a new starting point 1,000 times and chooses the best solution at the final step.

We assumed that we have 1,000 SNPs. We randomly decided the frequencies of the SNPs from the uniform distribution (0.3, 0.7). We generated 30 reference individuals (R) and one target individual t based on the frequencies. We calculated the distance vector v (squared Euclidean distances) between R and t.

To examine if the algorithm works as intended, we monitored SSE (the sum of squared errors between v and v', the distance vector of the candidate target sample) as the algorithm proceeded. As shown in the plot below, SSE decreased by orders of magnitudes as the algorithm steps advanced.

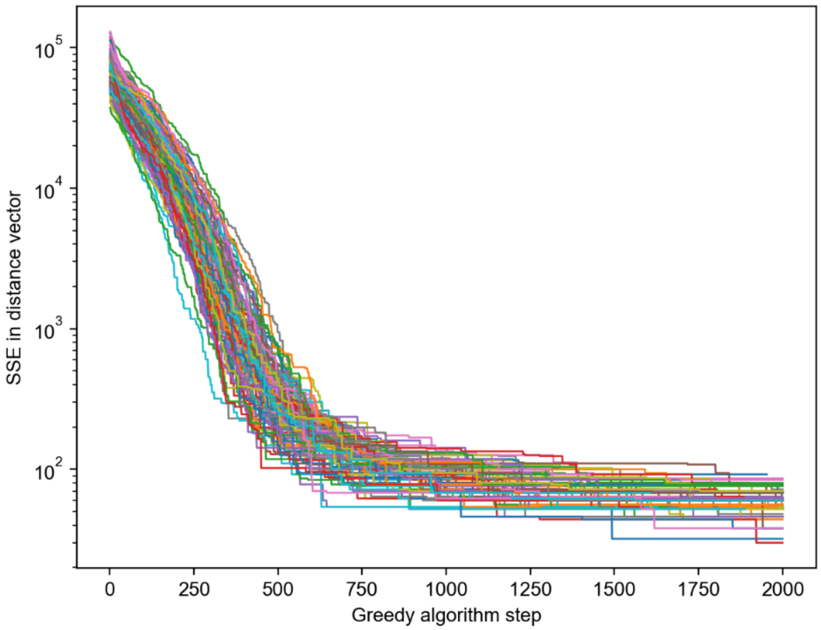

We then ran this algorithm 1,000 times to obtain 1,000 solutions. Note that each solution was the best selection among 1,000 candidate local optima. Given 1,000 solutions, we were able to measure overall accuracies of the prediction. For comparison, we generated another set of 1,000 solutions simply by random generation based on the allele frequencies.

We measured accuracy as the proportion of the correct allele count (0/1/2) in the data. Let $[X_{ij}]$ be 1000×1000 matrix where X_{ij} is 1 if the ith solution's jth SNP is correct and 0 otherwise. If we average each row of this matrix, we obtain the vector of per-individual accuracy. If we average each column of this matrix, we obtain the vector of per-SNP accuracy. Although the grand average of elements of each vector will be equal for both vectors, the two kinds of accuracies can give different interpretations. Our greedy algorithm works for each row (individual) to minimize the difference in distance vector. Thus, we can expect that the greedy algorithm may increase the per-individual accuracy to some degrees when we compare the greedy solutions to random solutions. The per-SNP accuracy is also of interest. Each SNP has a randomly set minor allele frequency, and the relative accuracy of the random solution may depend on the frequency. Moreover, it is important to evaluate if the greedy algorithm can give good accuracy for a single or a subset of SNPs.

We first evaluated the per-individual accuracy. The average per-individual accuracy was 39.4% in random samples and was 40.3% in greedy solutions (**Fig. S1a**). The t-test P-value was significant (P<1E-15), which means that the greedy algorithm was able to push the genetic contents toward the target sample. However, the small magnitude difference in accuracy (which is only 0.9%) shows that it is unlikely that the greedy algorithm can achieve 100% accuracy to recover the genome. Then, we measured the per-SNP accuracy. Unlike the per-individual accuracy, the t-test P-value was not significant (P=0.09). This shows that at the SNP level, the prediction by the greedy algorithm is not much better than the random prediction based on the allele frequency. The distributions are shown in **Fig. S1b**.

Finally, we checked if the risk score for a disease would be different between the two groups. We generated random weights of SNPs from the uniform distribution (0,1) and normalized them to have a sum of 1. We calculated the weighted average of the allele dosage (risk score) for individuals. When we examined the between-group difference of risk score between random samples and greedy solutions, *t*-test was significant (P=0.003). This was not surprising because the risk score can be considered per-individual information, and the per-individual accuracy showed difference above. However, the absolute magnitude of score was similar between the two groups; the mean of risk score was 0.989 in random samples and 0.986 in greedy solutions. This small difference shows that it is difficult to extract the risk information of an individual from the distance vector alone. The distributions are shown in **Fig. S1c** (where the blue dashed line indicates the risk score of the target sample).

5.3 Complexity analysis

Although simulations in the previous section showed that it is difficult to recover the gnomic data from distance vectors, the interpretation is not conclusive because we used a simple greedy algorithm. One may argue that if an opponent (who wants to breach privacy) develops a better algorithm, the genome can be revealed. Instead of implementing every possible algorithm as our opponent, which is impossible, we show a simple analysis on the complexity.

Assuming that we use 1,000 SNPs, our search space is of the size 3^{1000}. Apparently, this is a very large space. A widely used group of cryptographic hashing algorithms is a group called Secure Hashing Algorithm 2 (SHA-2), which includes the popular SHA-256 [13]. The complexity of SHA-256 is ~2^{255}. Thus, the search space of our problem is $\frac{3^{1000}}{2^{255}} = 10^{400}$ times bigger. Although the likelihood in our search space is not uniform due to the knowledge of allele frequencies, we can always reduce this skewness by selecting only common SNPs. Moreover, it is easy for us to increase the number of SNPs beyond 1,000. Overall, it is clear that the search space is large enough such that it is highly unlikely that the summarized information in distance vector can allow us to recover the exact solution.

6 Datasets used in the analysis

POPRES data

The samples we used in the PC map analysis were taken from the POPRES data set, which includes nearly 6,000 subjects of African-American, East Asian, South Asian, Mexican, and European origin [8]. The data were genotyped using the Affymetrix 500K SNP panel. For our analysis, we used a subsample of European individuals from the London Life Sciences Population (LOLIPOP) study [14], which comprises mainly European individuals sampled in London, and from the CoLaus study [15], which includes a broad set of European individuals sampled from Lausanne, Switzerland. **Table S2** summarizes the population distribution for the 1,387 individuals used in the final sample. POPRES data is accessible via dbGaP Study accession number phs000145.v4.p2 (https://www.ncbi.nlm.nih.gov/projects/gap/cgi-bin/study.cgi?study_id=phs000145.v4.p2)

WTCCC data

The WTCCC dataset includes ~3,000 shared controls and ~2,000 cases for each of seven diseases (including type 1 and type 2 diabetes, and Crohn's disease), with a total of ~14,000 cases on genotypes for up to 500,000 SNPs [3]. Access to WTCCC data is provided at European Genome-phenome Archive (EGA) with accession number EGAD00000000001 for 1958 British Birth Cohort and EGAD00000000008 for Type 1 Diabetes (T1D) samples (https://www.ebi.ac.uk/ega/datasets). The data were genotyped using the Affymetrix 500K SNP panel. We used ~1,500 controls from the 1958 British Birth Cohort and ~2,000 cases of type 1 diabetes. After a quality control process, 1,480 controls and 1,963 cases were used for our sample overlap detection simulation.

1000Genomes data

1000Genomes data [2] were used for our real data simulations. We used the phase 1 dataset which comprised whole genome sequencing and exome sequencing of 1,092 samples from 14 populations. The genotype data for 1000Genomes Phase 1 was downloaded from the webpage of PLINK 1.9 (http://www.cog-genomics.org/plink/1.9/resources). For the real-data-based simulation for sample overlap detection, 50 randomly selected samples were used as reference set. For the PC map analysis, 201 samples of the British (GBR), Tuscan (TSI), and Spanish (IBS) populations were used as a reference set to infer the spatial structure of the POPRES data. For ancestry estimation analysis, we used the phase 3 dataset of which the haplotype data were downloaded from ftp://ftp.1000genomes.ebi.ac.uk/vol1/ftp/release/20130502/.In the simulation of admixed samples from two populations, we used 302 samples of British (GBR), Tuscan (TSI), and Japanese (JPT). In the simulation of admixed samples from three populations, we used 304 samples of British (GBR), Chinese (CHS), and African (YRI).

7 Software implementation

We implemented our sample overlap detection method in a software package named `genomicgps`, which is coded in the Python programming language and is available at `https://github.com/hanlab-SNU/genomicgps`[16]. The software is available as a resource for the research community (Software DOI: 10.5281/zenodo.3255141[17]).

The software includes modules (1) to generate distance vectors given genomic data, (2) to calculate sample overlap detection statistic and P-value given distance vectors, and (3) to empirically determine an appropriate threshold of statistic that can distinguish the overlapping and unrelated pairs. The website also provides the Java codes for the greedy algorithm described in the section 5.2 and related simulation scripts.

Our software is efficiently implemented. In the WTCCC analysis in Section 2.6, for 3,496 samples (N=2000, K=50), it took 1.58 seconds to generate the distance vectors using a single core in the

computation server (Intel 2.10GHz CPU). Then, it only took 523 seconds to calculate the overlapping sample detection statistic for all 4,073,844 pairs.

References

1. The International HapMap Consortium. A haplotype map of the human genome. Nature. 2005;437:1299-1320.

2. The 1000 Genomes Project Consortium. A global reference for human genetic variation. Nature. 2015;526:68–74.

3. The Wellcome Trust Case Control Consortium. Genome-wide association study of 14,000 cases of seven common diseases and 3,000 shared controls. Nature. 2007;447:661-678.

4. Yang J, Lee SH, Goddard ME, Visscher PM. GCTA: a tool for genome-wide complex trait analysis. American journal of human genetics. 2011;88(1):76-82.

5. Patterson N, Price AL, Reich D. Population structure and eigenanalysis. PLoS genetics. 2006;2(12):e190-e190.

6. Novembre J, Johnson T, Bryc K, Kutalik Z, Boyko AR, Auton A, et al. Genes mirror geography within Europe. Nature. 2008;456(7218):98-101.

7. Mantilla-Gaviria I, Leonardi M, Galati G, Balbastre J. Localization algorithms for multilateration (MLAT) systems in airport surface surveillance. Signal, Image and Video Processing. 2014;9:1-10.

8. Nelson MR, Bryc K, King KS, Indap A, Boyko AR, Novembre J, et al. The Population Reference Sample, POPRES: a resource for population, disease, and pharmacological genetics research. American journal of human genetics. 2008;83(3):347-358.

9. Pritchard JK, Stephens M, Donnelly P. Inference of population structure using multilocus genotype data. Genetics. 2000;155(2):945-959.

10. Alexander DH, Novembre J, Lange K. Fast model-based estimation of ancestry in unrelated individuals. Genome research. 2009;19(9):1655-1664.

11. Su Z, Marchini J, Donnelly P. HAPGEN2: simulation of multiple disease SNPs. Bioinformatics (Oxford, England). 2011;27(16):2304-2305.

12. Hurwitz A. Ueber die angenäherte Darstellung der Irrationalzahlen durch rationale Brüche. Mathematische Annalen. 1891;39(2):279-284. http://eudml.org/doc/157573

13. National Institute of Standards and Technology. FIPS 180-2: Secure Hash Standard. Federal Information Processing Standards Publication 180-2, U.S. Department of Commerce. 2002. Available online: https://csrc.nist.gov/csrc/media/publications/fips/180/2/archive/2002-08-01/documents/fips180-2.pdf

14. Kooner JS, Chambers JC, Aguilar-Salinas CA, Hinds DA, Hyde CL, Warnes GR, et al. Genome-wide scan identifies variation in MLXIPL associated with plasma triglycerides. Nature Genetics. 2008;40(2):149-151.

15. Firmann M, Mayor V, Vidal PM, Bochud M, Pécoud A, Hayoz D, et al. The CoLaus study: a population-based study to investigate the epidemiology and genetic determinants of cardiovascular risk factors and metabolic syndrome. BMC cardiovascular disorders. 2008;8:6.

16. Kim K, Baik H, Jang CS, Roh JK, Eskin E, Han B. Genomic GPS: using genetic distance from individuals to public data for genomic analysis without disclosing personal genomes. Github. 2019. Available from: https://github.com/hanlab-SNU/GenomicGPS. Accessed 30 July 2019

17. Kim K, Baik H, Jang CS, Roh JK, Eskin E, Han B. Genomic GPS: using genetic distance from individuals to public data for genomic analysis without disclosing personal genomes. Zenodo. 2019; https://doi.org/10.5281/zenodo.3354656

Supplementary Tables

Table S1: False positive rate of the sample overlap detection statistic. We simulated 1,000,000 unrelated pairs of individuals. We used 1,000 loci and 20 reference individuals (N=1,000 and K=20). With a given threshold α, the false positive rate was estimated as the proportion of simulations with P-value $\leq \alpha$.

Threshold	False positive rate
0.05	0.049189
0.01	0.009932
0.005	0.00492
0.001	0.001017
0.0005	0.000527
0.0001	0.000108

Table S2: Population distribution of the POPRES dataset. The abbreviated population code was used in **Fig. 2a** and **Fig. 2b**.

Geoscheme for Europe	Population name	Population code	Total	Population total
Eastern Europe	Bulgaria	BG	2	
	The Czech Republic	CZ	11	
	Hungary	HU	19	
	Poland	PL	22	
	Romania	RO	14	
	Russia	RU	6	
	Slovakia	SK	1	
	Ukraine	UA	1	
	Turkey	TR	4	
	Cyprus	CY	4	84
Southern Europe	Albania	AL	3	
	Bosnia	BA	9	
	Montenegro	YG	41	
	Kosovo	KS	2	
	Macedonia	MK	4	
	Servia	RS	3	
	Croatia	HR	8	
	Greece	GR	8	
	Italy	IT	219	
	Portugal	PT	128	
	Slovenia	SI	2	
	Spain	ES	136	563
Western Europe	Austria	AT	14	
	Belgium	BE	43	
	France	FR	91	
	Germany	DE	71	
	Netherlands	NL	17	
	Switzerland	CH	222	458
Northern Europe	Denmark	DK	1	
	Finland	FI	1	
	Ireland	IE	61	
	Latvia	LV	1	
	Norway	NO	3	
	Sweden	SE	10	
	United Kingdom	UK	200	
	UK-Scotland	Sct	5	282
Total				1387

Supplementary Figures

Fig. S1: Comparison of greedy algorithm solutions and random solutions. We designed a greedy algorithm that tries to reconstruct the genotypes of a target sample given distance vector and reference data (see **Supplementary Note**). We simulated a target sample and reference individual data, assuming 30 reference individuals and 1,000 SNPs (K=30 and N=1,000) and applied the algorithm to the data. We also constructed random solutions based on allele frequencies. We compared the greedy and random solutions (1,000 solutions each) to the target sample genotypes and measured accuracy. **a,** Per-individual prediction accuracy. **b,** Per-SNP prediction accuracy. **c,** Per-individual risk score accuracy for a simulated set of risk weights of SNPs. The blue dash line is the risk of the target sample.

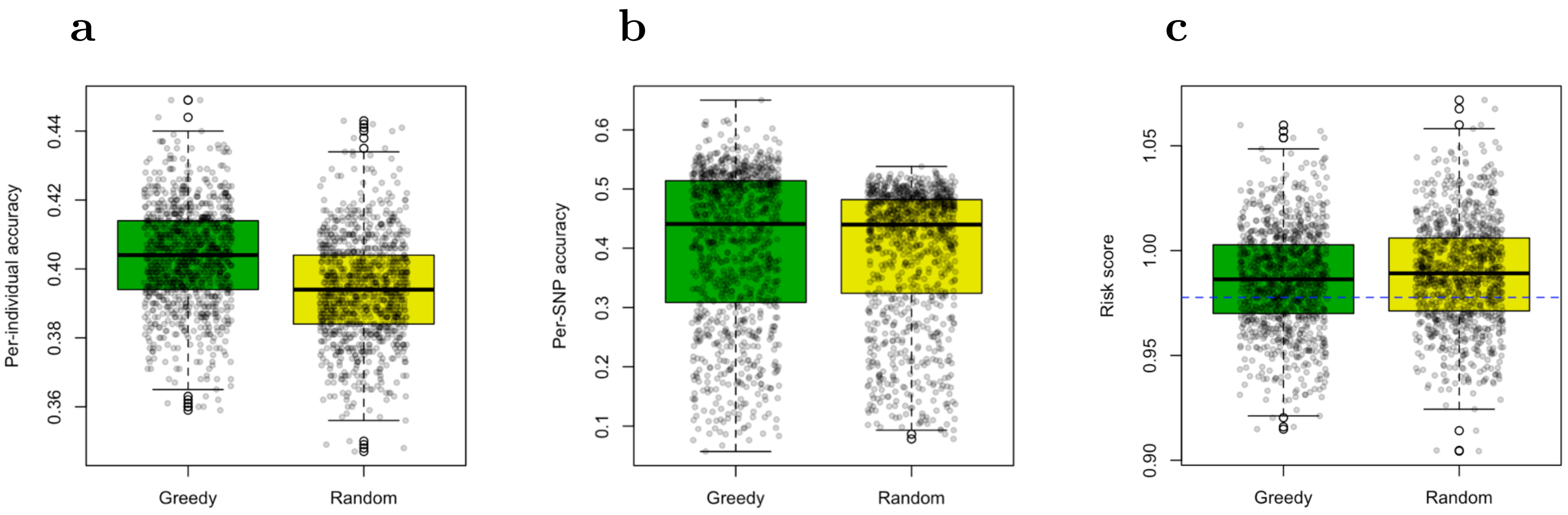

Fig. S2: The empirical distribution of the overlapping sample detection statistic. We show the histogram of 1,000,000 statistics simulated under the null hypothesis of unrelated pairs, assuming 20 reference individuals and 1,000 SNPs (K=20 and N=1,000). Blue line denotes the probability density function of the chi-square distribution with 20 degrees of freedom.

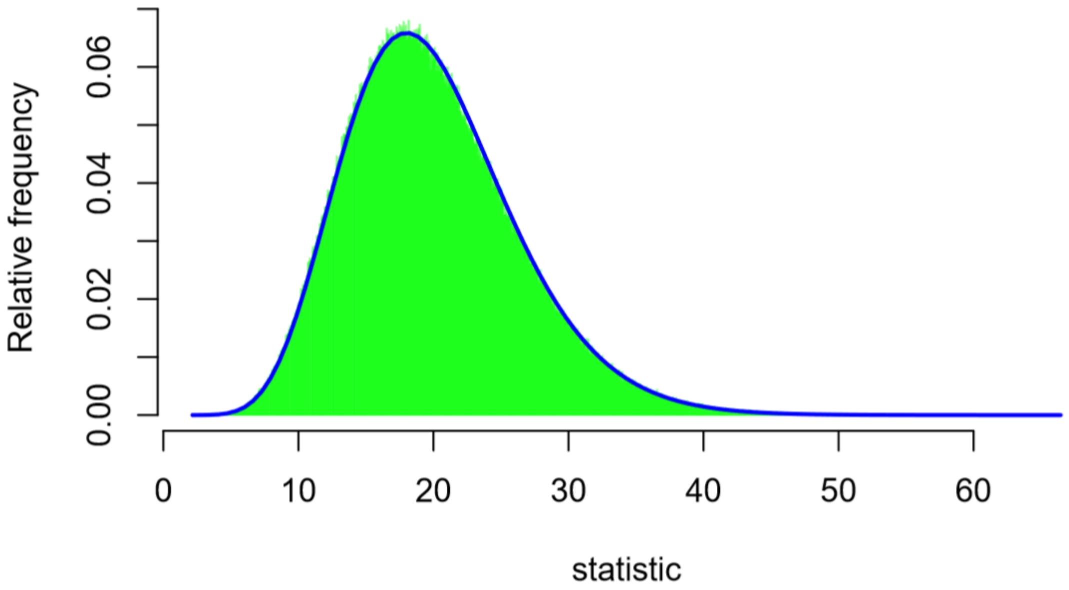

Fig. S3: Characteristics of the sample overlap detection method when using genetic relatedness as the distance measure. a, P-value distribution under the null hypothesis. 10,000 pairs were simulated under the null hypothesis where pairs were unrelated. Their distance vectors were calculated as the genetic relatedness to the 20 simulated reference individuals (N=500, K=20). X-axis is the P-value acquired from the lower tail of χ^2_{20} distribution. **b**, Density plot of the statistic under the null and the alternative hypotheses. 10,000 pairs were simulated under both hypotheses (null hypothesis: the pairs were unrelated, and alternative hypothesis: the pairs were sample overlap or identical twins). Blue bars show the density of statistics under the null hypothesis and red bars show the density of statistics under the alternative hypothesis.

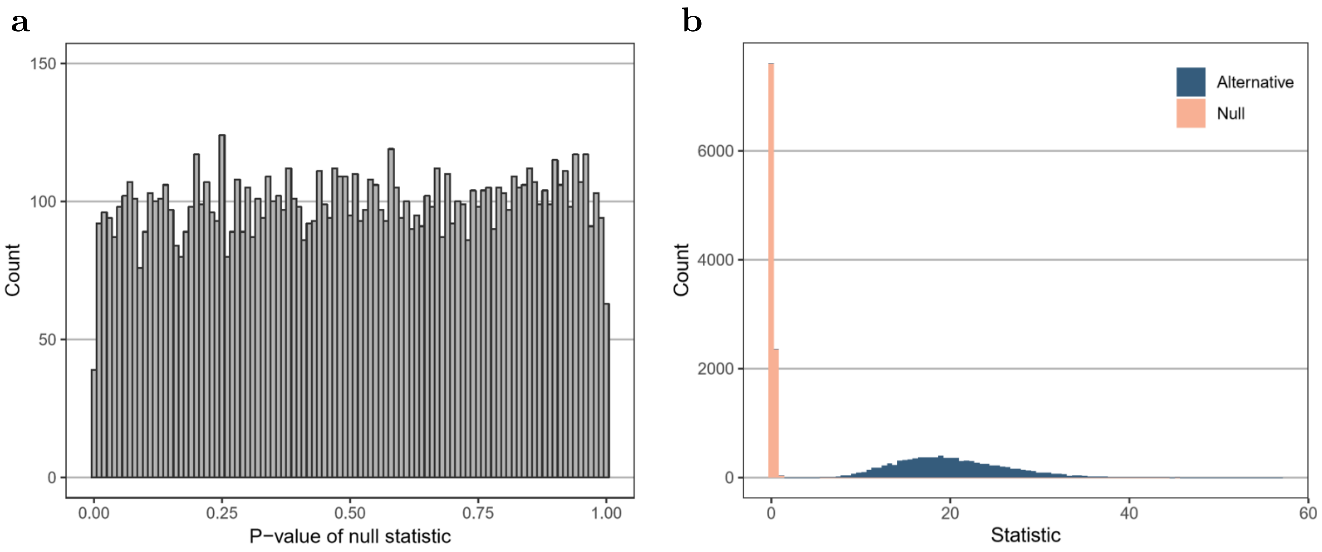

Fig. S4: Distribution of the sample overlap detection statistic for different degrees of relatives. We obtained the distribution of our sample overlap statistic under different relationships of pairs: sample overlap (or twins), 1st degree relatives, 2nd degree relatives, 3rd degree relatives, and unrelated pairs. We assumed 1,000 loci and 30 reference individuals (N=1000 and K=30). **a,** The density of statistic for differing degrees of relatives. **b,** The posterior probability of being in each category given the statistic, which was calculated as the probability density of the category divided by the sum of the densities of all categories. **c,** The proportion of correct assignment. This is the proportion of samples of a specific relationship that was correctly assigned to that relationship after determining the most likely relationship based on the posterior probability.

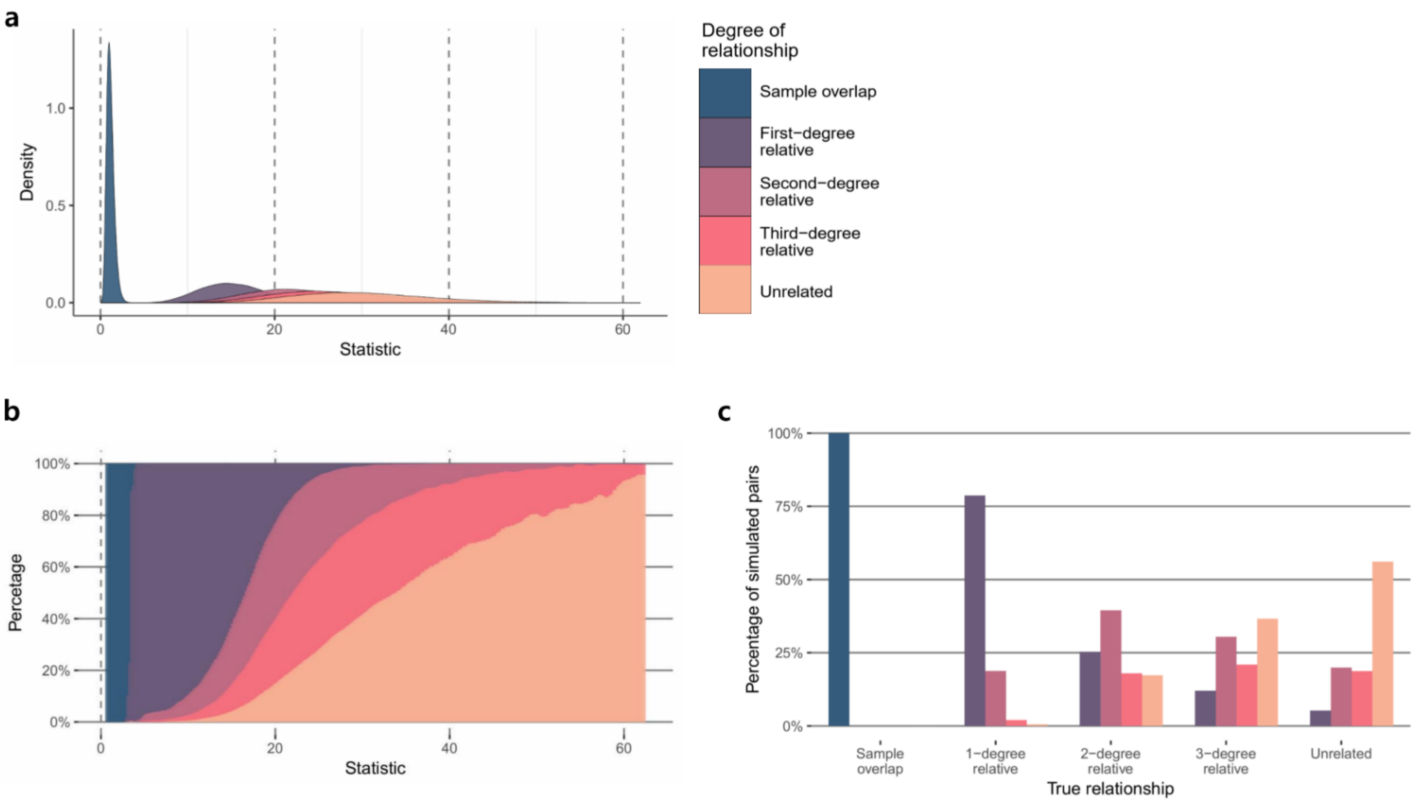

Fig. S5: Precision and recall of relationship prediction using sample overlap detection statistic. We simulated 100,000 pairs assuming each of 5 relationships. We assumed 1,000 loci and 30 reference individuals (N=1000 and K=30). We calculated their sample overlap detection statistics and predicted the most likely relationship based on the statistics. We measured performance of our predictions using 3 metrics; precision was measured as the proportion of true assignments out of total number of predicted assignments to a specific relationship, recall was measured as the proportion of correct assignments out of total simulated pairs of a specific true relationship, and F-measure was calculated as $2 \times \frac{precision \times recall}{precision + recall}$.

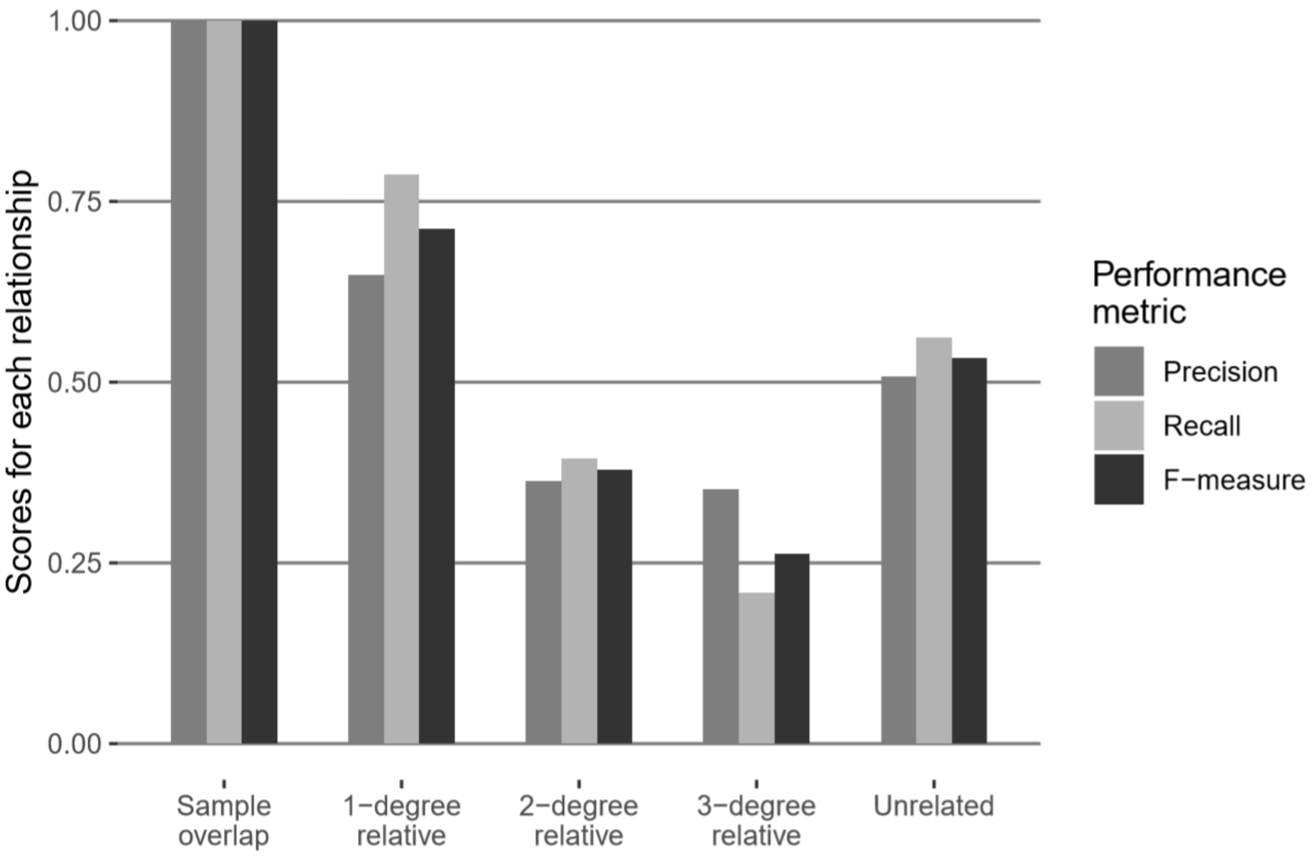

Fig. S6: Changes in the top two principal components of the reference data when we add one additional sample to data. a, In the POPRES analysis where we used 40% of the samples as reference, we plotted the reference samples' top two principal components. In the **Supplementary Note**, we call this space $\mathscr{P}$. **b,** To map a sample based on distance vector, we added the sample to the reference and regenerated the principal components. Because of this addition, the reference samples' positions were slightly distorted. In the **Supplementary Note**, we call this space $\mathscr{P}'$. **c,** Between subfigures **a** and **b**, we compared the location of the same reference datapoint and measured the difference (perturbation) in location. We repeated this comparison for all reference datapoints. The comparison was done after adjusting for the rotation to align the two figures as much as possible.

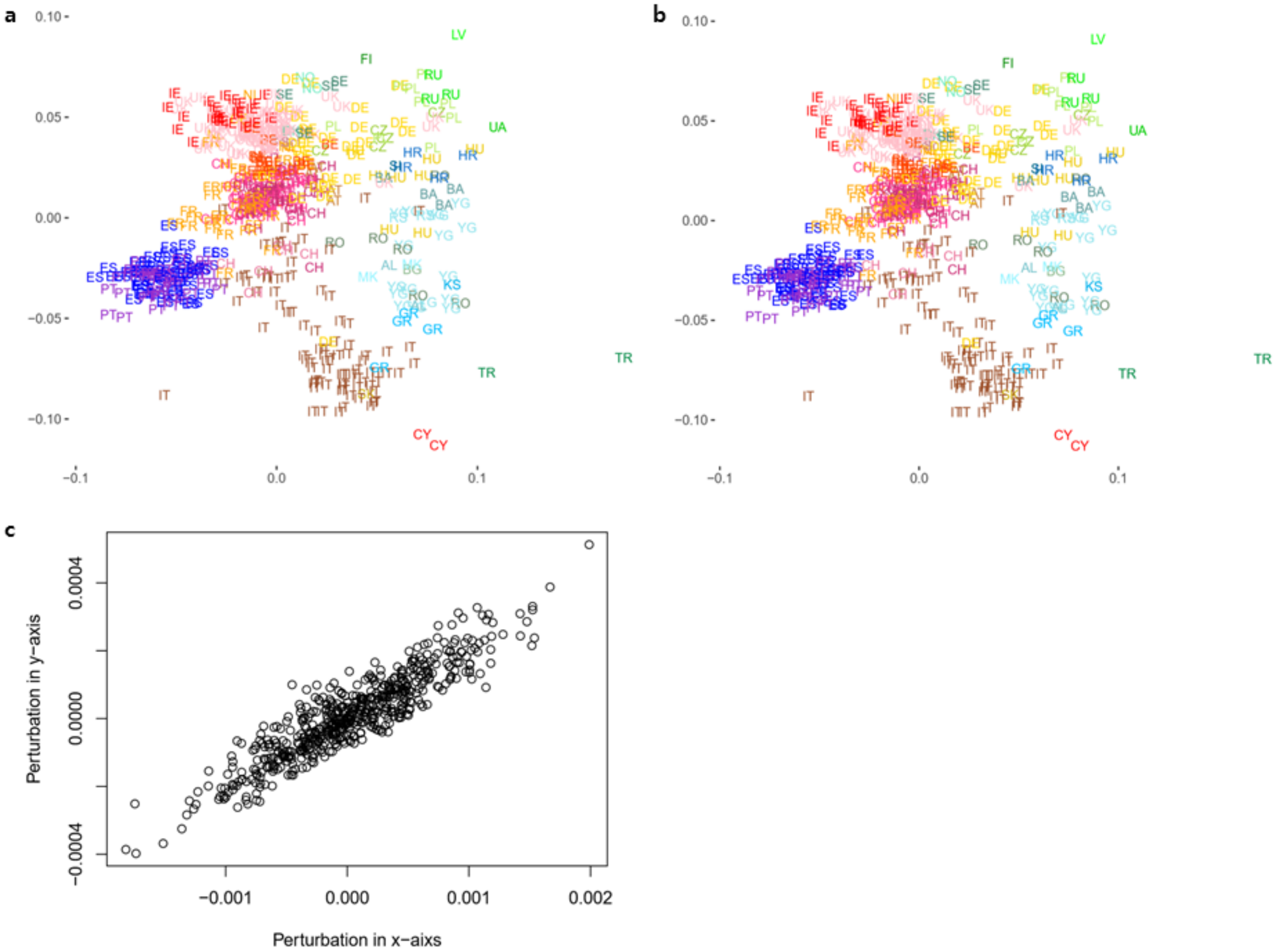

Fig. S7: Two-dimensional mapping of the Europeans in the POPRES data using distance vectors. We mapped the whole data of the POPRES individuals using the 1000Genomes data (201 European individuals from GBR, TSI, and IBS) as reference data to calculate distance vectors.

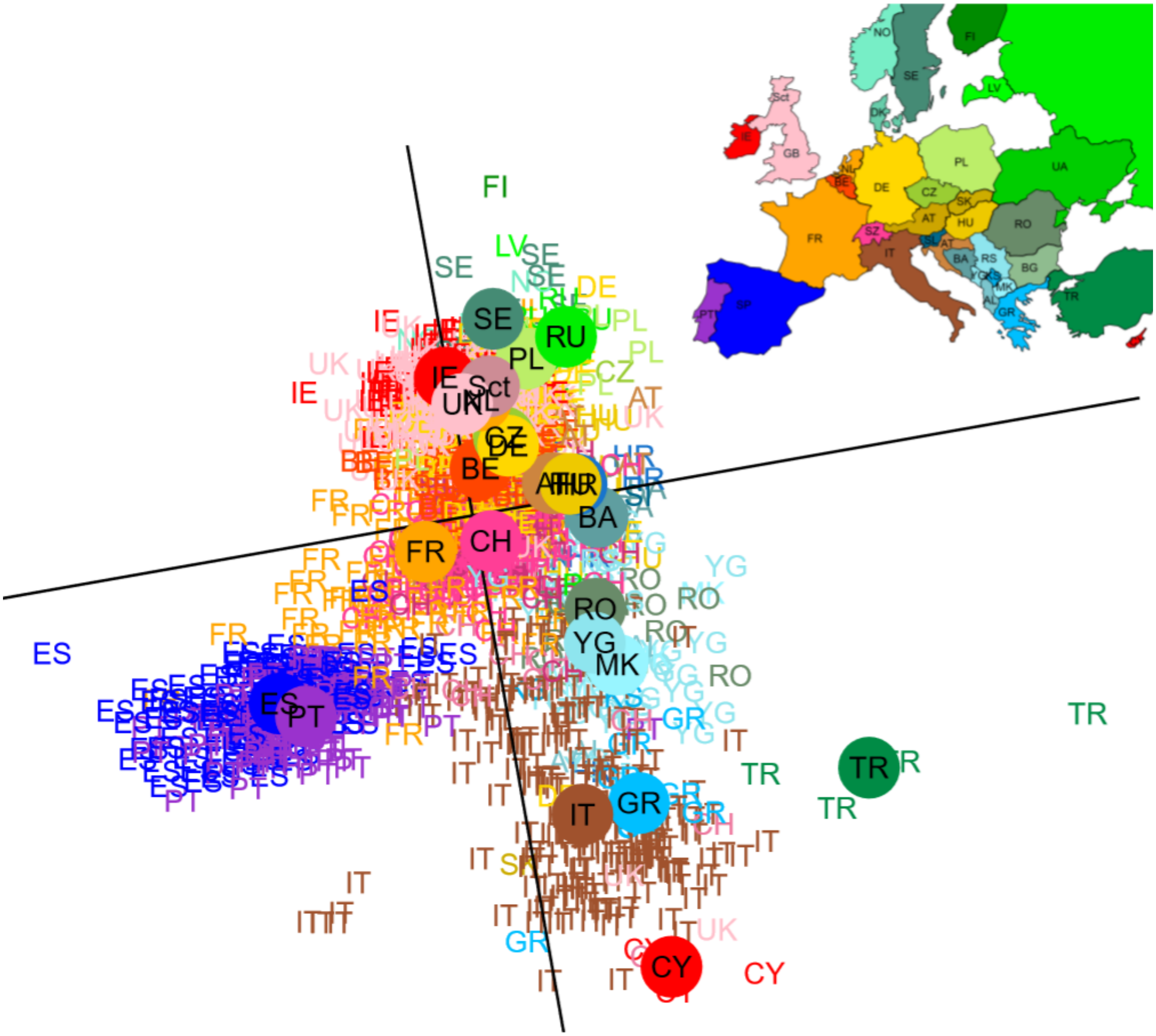

Fig. S8: *P*-value distribution of the sample overlap detection method for different *N* and *K*. We examined the validity of the asymptotic approximation of *P*-values for different numbers of loci (*N*) and reference individuals (*K*). A valid approximation will give us uniformly distributed *P*-values under the null hypothesis that individuals are unrelated. For each simulation, we generated 5,000 unrelated pairs. The ratio denotes *N* to *K* ratio, which is the number of SNPs divided by the number of references (N/K). At lower ratio (N/K<20), the distributions often showed peaks at one or both ends. At higher ratio (N/K>20), the distributions were closer to being uniform.

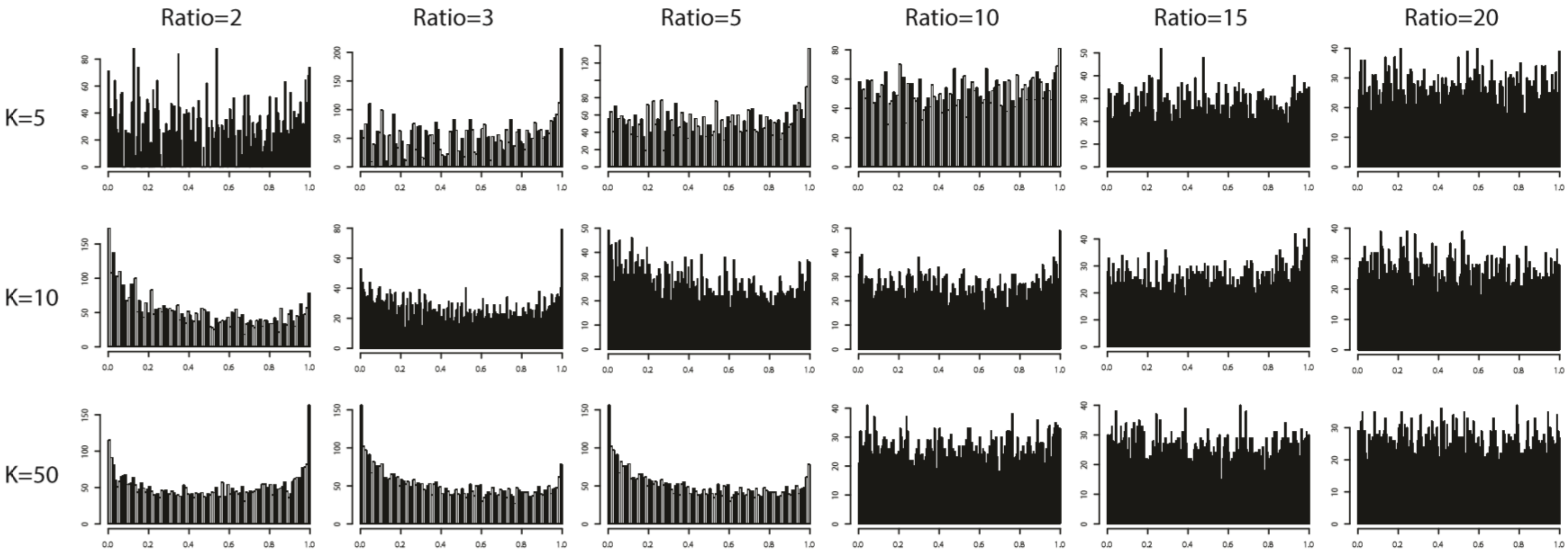

Fig. S9: *P*-values of the sample overlap detection method for unrelated and overlapping samples in an additional real-data-based simulation. To avoid possible sample selection bias in the real-data-based simulations using the WTCCC data described in Section 2.6, we performed an additional analysis to select 1,000 unrelated pairs and 1,000 overlapping pairs from the same dataset. The p-values of our overlapping sample detection method were distinct for the two groups, showing that the results in **Fig. 1f** were not driven by the bias.

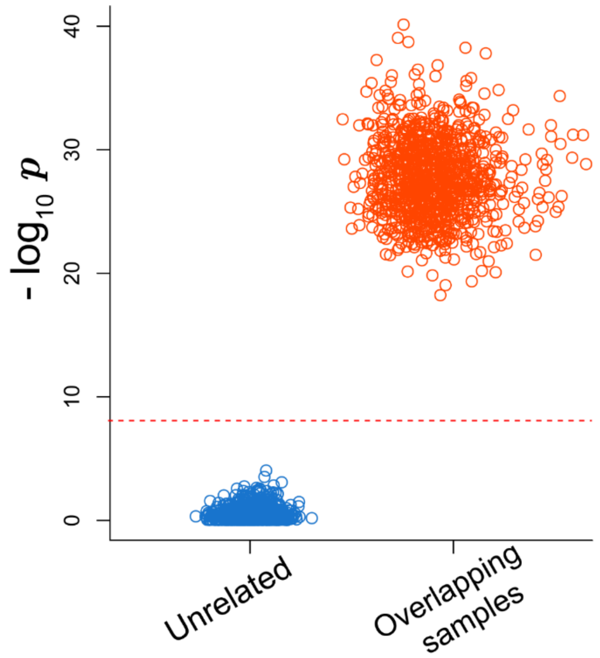

Fig. S10: Two-dimensional mapping of the Europeans in the POPRES data for varying numbers of variants. In the same analysis as in **Fig. 2a**, we gradually decreased the number of SNPs (N) by random subsampling.

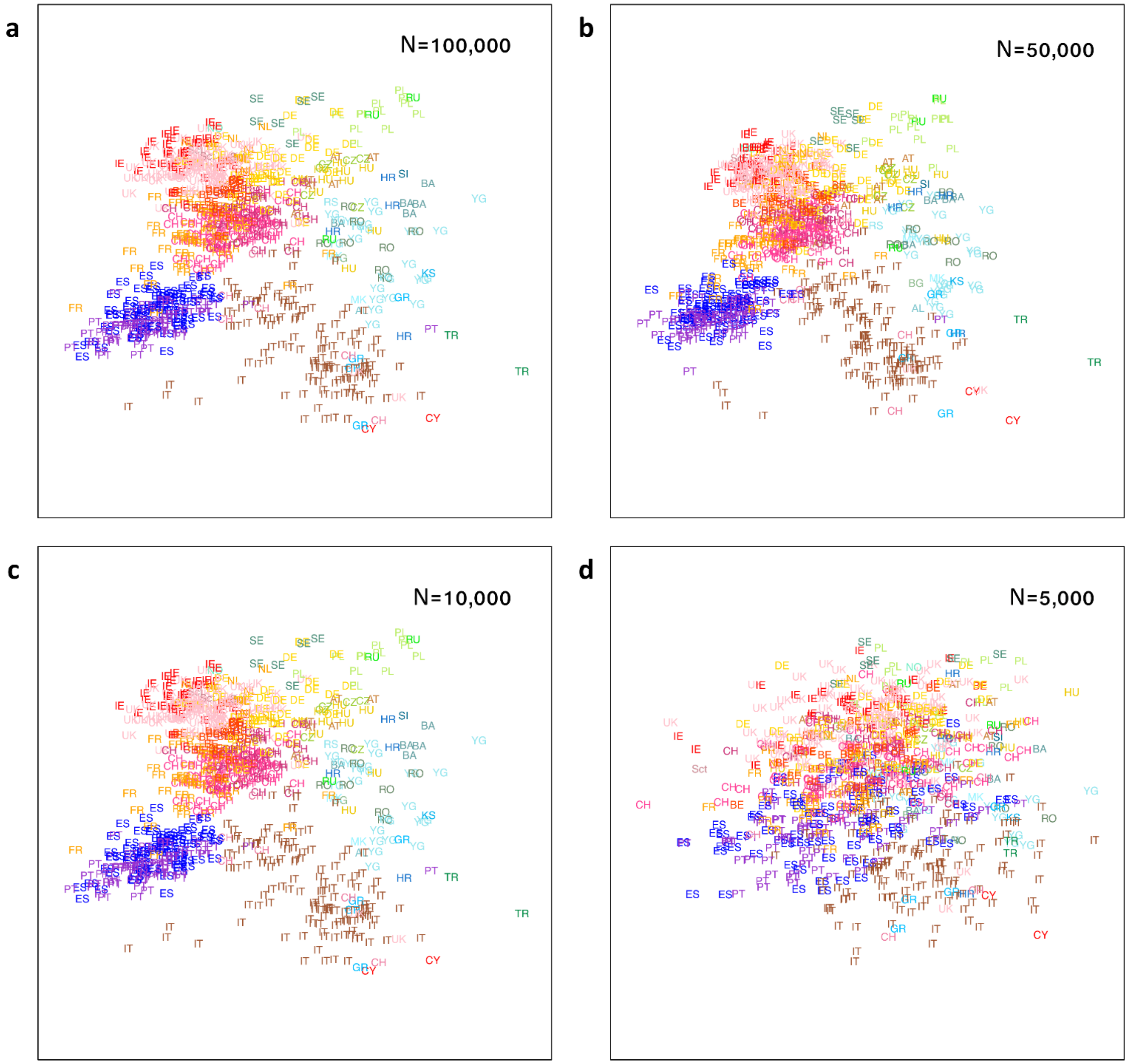

Fig. S11: Two-dimensional PC map of the samples used in the two-population ancestry estimation. Given the two reference populations, we used the subset of each population to generate the admixed samples and used the rest as the reference data for our method (denoted as diamonds here). To estimate the ancestry proportion of a sample, the PC1 distance was measured from the sample to each reference cluster, of which the inverse proportions were our estimates. **a,** The two populations were distant (GBR and JPT). **b,** The two populations were close (GBR and TSI).

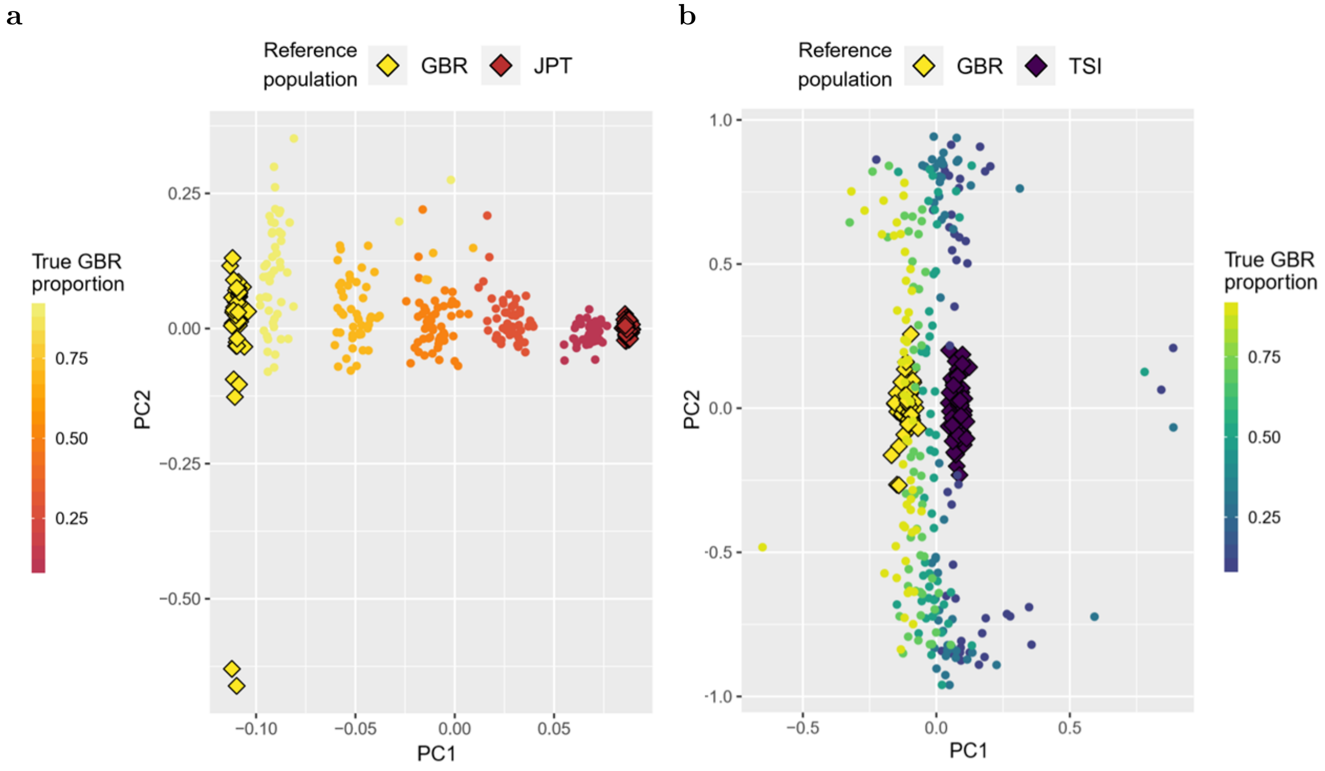

Fig. S12: Two-dimensional PC map of the samples used in the three-population ancestry estimation. Given the three reference populations, we used the subset of each population to generate the admixed samples and used the rest as the reference data for our method (denoted as diamonds here). To estimate the ancestry proportion of a sample, the Euclidean distance in the PC1-PC2 space was measured from the sample to each reference cluster, of which the inverse proportions were our estimates. **a,** True proportion of CHS ancestry is colored. **b,** True proportion of GBR ancestry is colored. **c,** True proportion of YRI ancestry is colored.

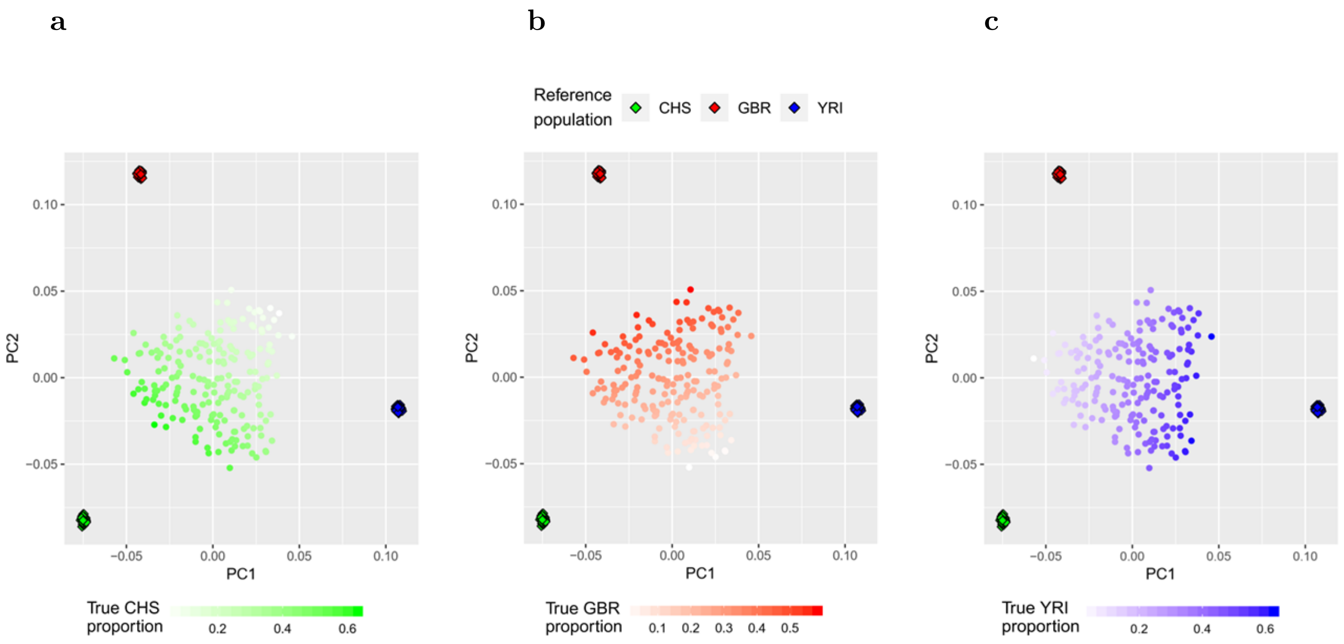

Supplement: Supplementary file 1 — Supplementary Note, Tables S1, S2 and Figures. S1-S12. (PDF 3657 kb) [file 13059_2019_1792_MOESM1_ESM.pdf]
